# Targeting the SOX9/TIMP1 Axis with iRGD‐Conjugated Nanoplatform Enhances Dendritic Cell Function and Photodynamic Immunotherapy in Gastric Cancer

**DOI:** 10.1002/advs.202510500

**Published:** 2025-11-21

**Authors:** Banghua Zhong, Yijie Zhang, Honghu Wang, Lili Guo

**Affiliations:** ^1^ Department of Gastrointestinal Surgery The First Hospital of China Medical University Shenyang 110001 China; ^2^ Department of Hepatobiliary and Organ Transplantation The First Affiliated Hospital of China Medical University Shenyang 110001 China; ^3^ Department of Surgical Oncology The First Affiliated Hospital of China Medical University Shenyang 110001 China; ^4^ Department of Anesthesiology The First Hospital of China Medical University Shenyang 110001 China

**Keywords:** dendritic cell maturation, gastric cancer immunotherapy, photodynamic therapy, SOX9/TIMP1 signaling axis, targeted nanoparticles

## Abstract

Gastric cancer (GC) remains a therapeutic challenge due to its immunosuppressive microenvironment. This study reveals that the SOX9/TIMP1/FAK/PI3K axis impedes dendritic cell (DC) maturation and antitumor immunity in GC, using integrated single‐cell RNA sequencing (scRNA‐seq) and spatial transcriptomics (ST‐seq) analysis. Irgd‐conjugated poly(lactic‐co‐glycolic acid) (PLGA) nanoparticles (NPs) co‐loaded with small interfering RNA targeting SOX9 (si‐SOX9), the photosensitizer chlorin e6 (Ce6), and L‐arginine (L‐Arg) (termed iRGD NPs@si‐SOX9/CL) are developed to simultaneously disrupt this immunosuppressive axis and amplify photodynamic therapy (PDT). In vitro, these NPs enhance cellular uptake and lysosomal escape, suppressing SOX9 expression and generating reactive oxygen species (ROS) and nitric oxide (NO) upon near‐infrared (NIR) irradiation. This dual action significantly inhibits GC cell proliferation, migration, and invasion while promoting DC maturation (evidenced by elevated CD80/CD86 expression) and CD8⁺ T‐cell activation. In vivo, iRGD modification boosts tumor accumulation of NPs, and NP‐mediated PDT synergized with SOX9 silencing to suppress tumor growth in GC xenografts. Mechanistically, NPs@si‐SOX9/CL blocks SOX9‐driven transcriptional activation of TIMP1, reversing FAK/PI3K signaling and restoring DC function. Consequently, tumor‐infiltrating mature DCs and cytotoxic T lymphocytes increase significantly, reshaping the immunosuppressive niche. This nanoplatform offers a promising strategy to augment GC immunotherapy by precisely targeting tumor‐intrinsic immunosuppression and potentiating PDT efficacy.

## Graphical abstract

This study identifies the SOX9/TIMP1 axis as a key regulator of immune evasion in gastric cancer. iRGD‐conjugated nanoparticles co‐delivering si‐SOX9, Ce6, and L‐Arg are developed to synergize with photodynamic therapy. The nanoplatform suppresses tumor growth, promotes dendritic cell maturation, and enhances CD8^+^ T‐cell activation, offering a promising multimodal immunotherapy strategy for gastric cancer.

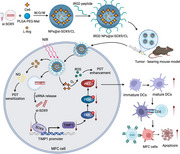

## 1 Introduction

Gastric cancer (GC) is one of the most common malignant tumors worldwide. Recent global cancer statistics rank GC as one of the leading cancers in both incidence and mortality.^[^ ^1^, ^2^ ^]^ Although diagnostic and therapeutic technologies have advanced considerably, including improvements in endoscopy, refinement of surgical techniques, and the development of chemotherapy and targeted agents, the five‐year survival rate of GC patients remains unsatisfactory.^[^ ^3^ ^]^ Treatment of advanced GC poses particular challenges, as the majority of patients are diagnosed at late stages with widespread metastasis. Many cases cannot undergo curative resection because of tumor invasion into adjacent organs.^[^ ^4^, ^5^ ^]^ These limitations highlight the urgent need for novel therapeutic approaches, especially strategies that can effectively activate the immune system to suppress tumor progression.

In tumor immunology, dendritic cells (DCs) serve as key antigen‐presenting cells, and the expression levels of surface molecules directly influence their activation state and ability to stimulate effector T cells.^[^ ^6^, ^7^ ^]^ Upregulation of these molecules enhances antigen uptake and processing by DCs and strengthens interactions with T cells, thereby promoting T‐cell activation and proliferation.^[^ ^8^, ^9^ ^]^ Previous studies have shown that pan‐PI3K inhibition markedly increases the expression of CD80, CD86, MHC‐I, and MHC‐II in DCs. During antigen presentation, DCs with higher levels of costimulatory molecules such as CD80 and CD86 exhibit a stronger capacity to activate T cells.^[^ ^10^ ^]^ Thus, modulating DC activation offers a promising approach for improving cancer immunotherapy.

Photodynamic therapy (PDT) is a promising minimally invasive approach for tumor treatment that induces apoptosis by converting O_2_ molecules into reactive oxygen species (ROS) with high efficiency.^[^ ^11^ ^]^ L‐arginine (L‐Arg), a natural and biocompatible nitric oxide (NO) precursor, serves as the substrate for nitric oxide synthase (NOS) to generate NO. Compared with direct NO gas delivery or synthetic NO donors (e.g., nitrates, S‐nitrosothiols), L‐Arg produces NO through endogenous enzymatic pathways, enabling slower and more controllable release while avoiding excessive NO‐induced cytotoxicity and oxidative stress.^[^ ^12^ ^]^ In the tumor microenvironment (TME), L‐Arg can be catalyzed by inducible NOS (iNOS) and activated under tumor‐specific conditions (e.g., high H_2_O_2_ levels) to achieve localized NO release, thereby promoting vasodilation, alleviating tumor hypoxia, improving ROS generation efficiency in PDT, and enhancing tumoricidal effects. Compared with other NO donors, L‐Arg more closely aligns with the characteristics of the TME, achieving synergistic therapeutic outcomes.^[^ ^13^ ^]^ However, the efficacy of PDT remains constrained by the selection of photosensitizers and the efficiency of their delivery systems.^[^ ^14^ ^]^ Advances in nanotechnology have provided new strategies for photosensitizer delivery. Encapsulation of photosensitizers in nanoparticles (NPs) enhances tumor accumulation, reduces off‐target toxicity, and increases tumor cell specificity through surface modifications, thereby improving therapeutic efficacy.^[^ ^15^, ^16^, ^17^ ^]^

In recent years, single‐cell RNA sequencing (scRNA‐Seq) and spatial transcriptomic sequencing (ST‐Seq) have become powerful tools for characterizing the TME. These complementary approaches enable high‐resolution profiling of cellular heterogeneity and spatial organization, thereby providing insights into intercellular communication and immune evasion mechanisms.^[^ ^18^, ^19^, ^20^, ^21^ ^]^ In the present study, scRNA‐seq and ST‐seq were applied to delineate the immune landscape of GC, thereby generating mechanistic evidence to support the proposed therapeutic strategy.

To improve the effectiveness of immunotherapy for GC, an innovative nanoplatform was developed that integrates gene silencing with PDT. Specifically, PLGA NPs co‐loaded with small interfering RNA against SOX9 (si‐SOX9), Ce6, and L‐Arg (NPs@si‐SOX9/CL) were engineered to target the SOX9/TIMP1/FAK/PI3K signaling axis. This strategy not only inhibits immune evasion by GC cells but also improves DC activation and stimulates effective anti‐tumor T cell response. By combining this therapeutic approach with high‐resolution transcriptomic tools, we systematically investigated the molecular mechanisms underlying its efficacy.

This study aims to systematically analyze the mechanisms by which NPs@si‐SOX9/CL regulate the SOX9/TIMP1/FAK/PI3K axis in PDT and their impact on DC activation and enhancement of the immune response in GC. By focusing on this axis, the approach seeks to strengthen DC‐mediated antitumor immunity within the GC microenvironment and provide a promising basis for clinical translation.

## 2 Results

### 2.1 Enhanced Interaction Between Cancer Cells and DCs in GC Revealed by scRNA‐seq and ST‐seq Analysis

**Figure 1:**
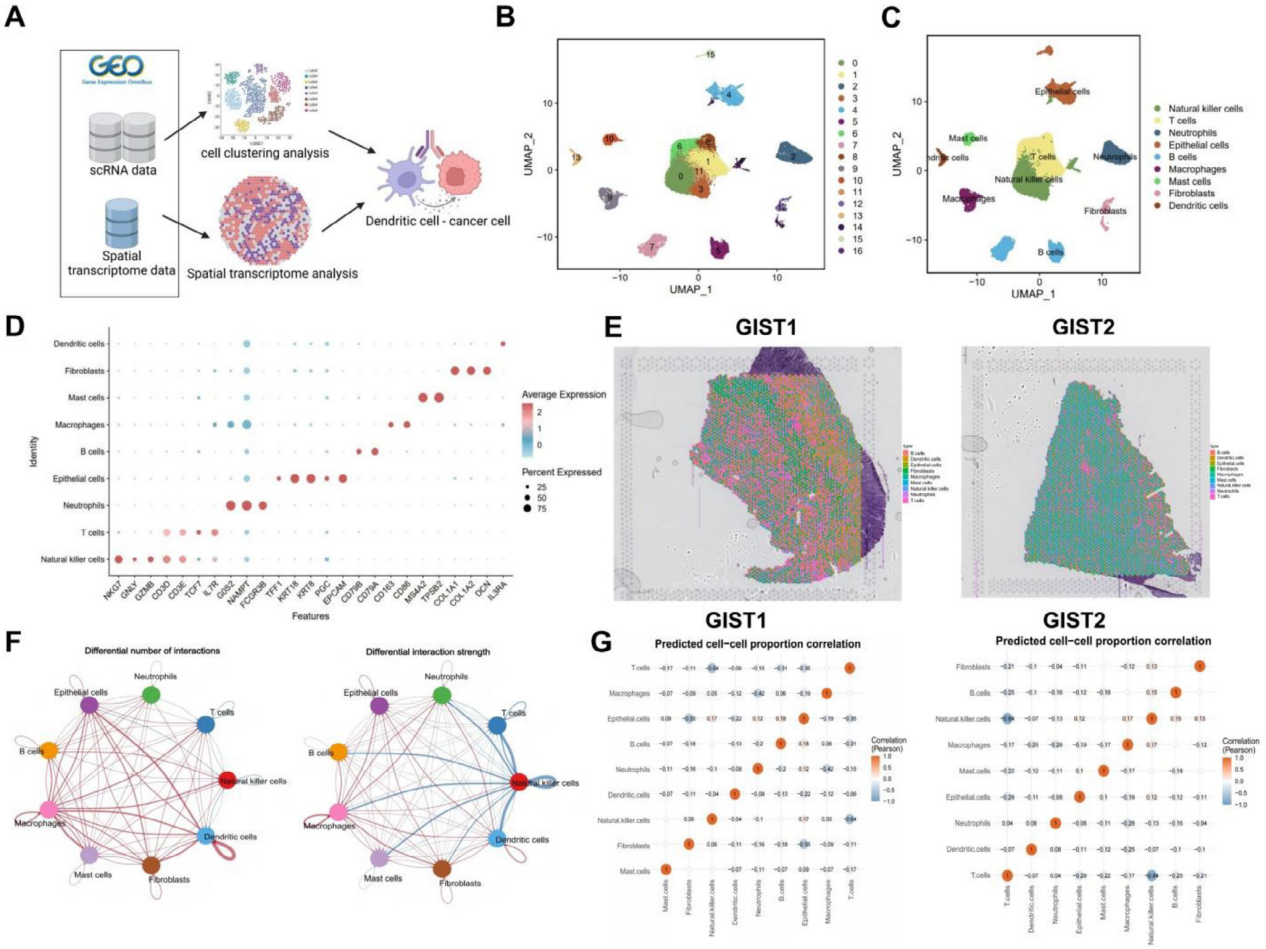
ScRNA‐seq and ST‐seq data analyses reveal the cellular distribution and interactions in GC. Note: A) Workflow schematic showing the retrieval of scRNA‐seq and ST‐seq data from the GEO database, followed by cell clustering analysis, spatial transcriptomics analysis, and exploration of DC‐cancer cell interactions; B) UMAP algorithm clustering analysis of integrated scRNA‐seq data, displaying the distribution of 17 cell clusters; adjacent normal tissue (Normal): n = 2, GC tissue (Tumor): n = 4; C) Nine cell types annotated based on literature and cell marker genes; D) Dot plot of marker gene expression across cell types, showing the intensity and proportion of expression of cell type‐specific marker genes; E) ST‐seq data analysis showing the spatial distribution of different cell types in GC tumor tissue, n = 2, with each spot's cell distribution shown in pie charts; F) Circos plot of changes in cell communication between Tumor and Normal groups, with red lines indicating increased communication in Tumor cells and blue lines indicating decreased communication, left chart line thickness represents the number of pathways, right chart line thickness represents interaction strength; G) SPOTlight analysis showing a heatmap of the correlation of various cells in GC tissue.

To investigate cellular interactions in GC, we obtained scRNA‐seq and ST‐seq datasets from the Gene Expression Omnibus (GEO) database and analyzed them for cell‐type expression and spatial distribution (**Figure** **1**A).

The GSE163558 and GSE184198 datasets were integrated using the Seurat package. Following quality control, batch effects were corrected with the Harmony package, and nonlinear dimensionality reduction was performed with UMAP on the top 30 principal components (PCs). Clustering analysis identified 17 distinct clusters (Figure 1B). Cell annotation was carried out by referencing lineage‐specific marker genes from the literature and the CellMarker database, resulting in the identification of nine major cell types: epithelial cells (clusters 4 and 15), fibroblasts (clusters 12 and 16), DCs (cluster 13), mast cells (cluster 10), natural killer cells (clusters 0, 3, and 14), macrophages (cluster 9), neutrophils (cluster 2), B cells (clusters 5 and 7), and T cells (clusters 1, 6, 8, and 11) (Figure 1C). A dot plot was used to visualize the expression of representative marker genes across these cell types (Figure 1D).

To characterize spatial patterns of cell distribution in GC tissues, tumor sections from two patients in the ST‐seq dataset GSE245908 were analyzed. The ST data were integrated and normalized with the Seurat package (Figure S1A,B, Supporting Information). Highly variable genes were selected for principal component analysis (PCA), followed by UMAP for dimensionality reduction and clustering (Figure S1C,D, Supporting Information). Spatial annotation of cell types was inferred based on the overlap between ST‐seq genes and scRNA‐seq–defined cell‐type markers (Figure 1E).

Cell–cell communication was further explored using the CellChat package in R. Compared with adjacent normal tissues, GC tissues exhibited stronger interactions between epithelial cells (cancer cells) and immune cells, with the most notable enhancement observed between epithelial cells and DCs (Figure 1F; Figure S2, Supporting Information). Using the SPOTlight package in *R*, the spatial distribution of epithelial and dendritic cells was mapped in tumor tissues from two GC patients (Figure S1E,F, Supporting Information). A correlation heatmap revealed a significant negative correlation between the proportions of DCs and epithelial cells within tumor tissues (Figure 1G). DCs play a crucial role in antigen presentation by capturing, processing, and presenting tumor‐associated antigens to T cells, thereby initiating immune responses. Previous studies have shown that a reduction in intratumoral DCs or alterations in their immunoregulatory molecules can impair antitumor T‐cell responses.^[^ ^22^, ^23^ ^]^

Based on these findings, we hypothesize that GC cells may escape immune surveillance through enhanced interactions with DCs.

### 2.2 Transcriptomic Analysis Reveals *SOX9*/*TIMP1* Mediated DC Activation in GC Progression

**Figure 2:**
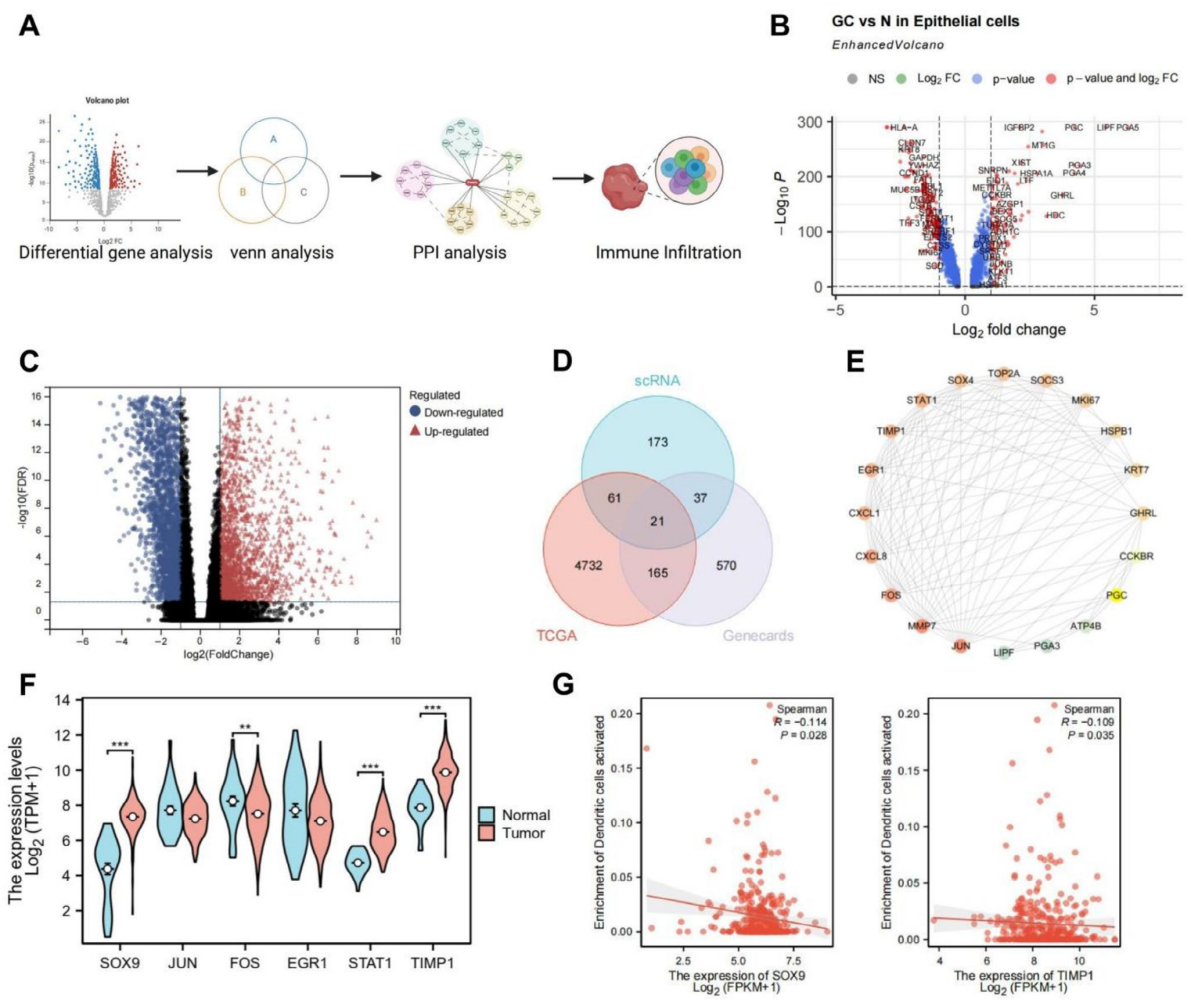
Transcriptomic analysis reveals the role of SOX9/TIMP1‐mediated DC activation in GC progression. Note: A) Bioinformatics analysis workflow schematic, showing differential gene analysis, intersecting gene filtering, protein interaction analysis, and immune infiltration analysis; B) Volcano plot of differential genes in GC epithelial cells from scRNA‐seq data analysis; C) Volcano plot of differential genes from TCGA database GC transcriptomic data, Normal: n=32, Tumor: n = 375; D) Venn diagram of intersecting disease‐related genes from scRNA‐seq, TCGA, and the GeneCards database; E) PPI analysis and network diagram based on 21 intersecting genes; F) Expression of candidate target genes (*JUN*, *SOX9*, *FOS*, *EGR1*, *STAT1*, and *TIMP1*) in GC tissues, Normal: n = 32, Tumor: n = 375, ^**^ *p*<0.01, ^***^ *p*<0.001; *G*) Correlation analysis of *SOX9* and *TIMP1* expression with mature DC infiltration.

Through integrated transcriptomic sequencing and immune infiltration analysis, we investigated how interactions between tumor cells and DCs regulate GC progression (**Figure** **2**A). Initially, gene expression data for epithelial cells (cancer cells) were extracted from scRNA‐seq, revealing 292 differentially expressed genes (DEGs) (Figure 2B). Differential analysis of GC transcriptomic data from The Cancer Genome Atlas (TCGA) identified 4,979 DEGs (Figure 2C). Intersection of these with 793 GC‐related genes from the GeneCards database yielded 21 overlapping genes (Figure 2D). Kyoto Encyclopedia of Genes and Genomes (KEGG) enrichment analysis indicated that these genes were predominantly enriched in immune‐related pathways, including IL‐17 signaling, Toll‐like receptor signaling, TNF signaling, and the PD‐L1/PD‐1 checkpoint pathway in cancer (Figure S3A, Supporting Information).

Protein–protein interaction (PPI) analysis of the 21 overlapping genes was performed and visualized using a Degree‐based network (Figure 2E). Least Absolute Shrinkage and Selection Operator (LASSO) regression further reduced the set to 15 disease‐related genes (Figure S3B, Supporting Information). Among these, six genes (*JUN*, *SOX9*, *FOS*, *EGR1*, *STAT1*, and *TIMP1*) were highlighted based on the Degree value, with differential expression shown in Figure 2F. Notably, *SOX9*, *STAT1*, and *TIMP1* were significantly overexpressed in GC tissues, whereas *FOS* was underexpressed. Previous studies have also reported high expression of *SOX9*, *FOS*, *STAT1*, and *TIMP1* in GC tissues.^[^ ^24^, ^25^, ^26^, ^27^ ^]^ Kaplan–Meier survival analysis further demonstrated that elevated expression of *SOX9* and *TIMP1* predicted poor prognosis, while higher *STAT1* expression was associated with better outcomes (Figure S3C, Supporting Information). Thus, *SOX9* and *TIMP1* were identified as key regulators of GC progression. Predictive analysis using the JASPER website identified multiple *SOX9* binding sites within the *TIMP1* promoter region (Table S1, Supporting Information).

To explore the impact of *SOX9* and *TIMP1* expression on DC infiltration and function in GC, immune infiltration analysis revealed a negative correlation between their expression levels and the infiltration of mature DCs (Figure 2G; Figure S3D, Supporting Information).

These results suggest that *SOX9* may promote GC progression by transcriptionally activating *TIMP1* to inhibit dendritic cell maturation.

### 2.3 Transcriptional Activation of *TIMP1* by SOX9 in GC Cells

**Figure 3:**
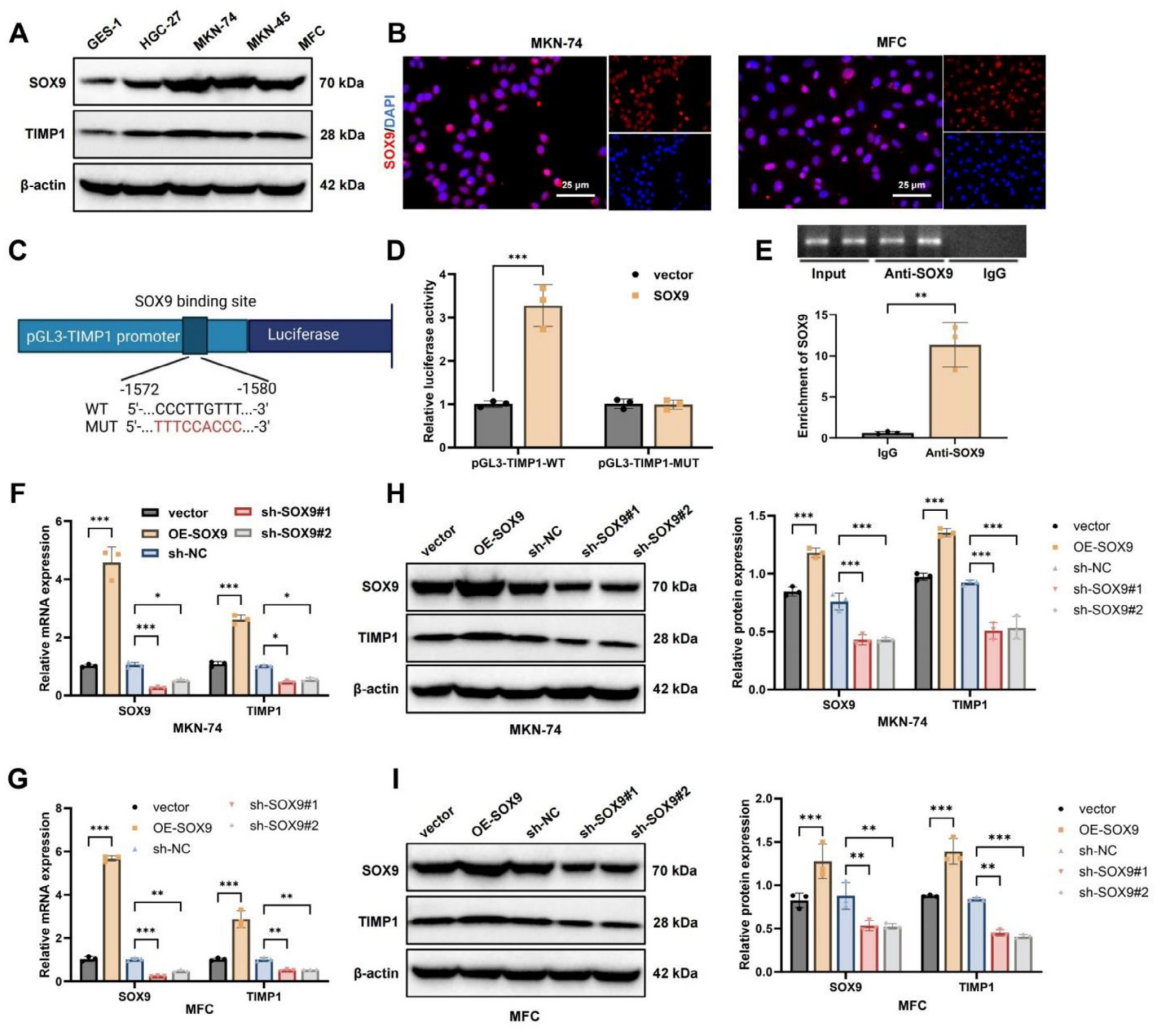
Regulation of *TIMP1* transcription and expression by SOX9. Note: A) Western Blot analysis of SOX9 and TIMP1 protein expression in various GC cell lines; B) Immunofluorescence staining analyzing the localization of SOX9 in MKN‐74 and MFC cells (Scale bars = 25 µm); C) Schematic of the *TIMP1* promoter and its mutant sequences showing SOX9 binding sites; D) Dual‐luciferase assay validating the regulatory effect of SOX9 on *TIMP1* promoter activity; E) ChIP assay detecting the enrichment of SOX9 at the *TIMP1* promoter region; F,G) RT‐qPCR assessing mRNA levels of *SOX9* and *TIMP1* in MKN‐74 and MFC cells post‐overexpression or knockdown of SOX9; H,I) Western Blot analysis of SOX9 and TIMP1 protein levels in MKN‐74 and MFC cells following overexpression or knockdown of SOX9. ^*^ *p*<0.05, ^**^ *p*<0.01, ^***^ *p*<0.001, experiments repeated three times.

To further clarify the regulatory role of SOX9 in *TIMP1* transcription, we initially examined the protein expression of SOX9 and *TIMP1* across various GC cell lines. Compared with GES‐1 normal gastric epithelial cells, both proteins were upregulated in human GC cell lines, with the highest expression detected in MKN‐74 cells; similar expression patterns were observed in the murine GC cell line, mouse forestomach carcinoma (MFC) (**Figure** **3**A). MKN‐74 cells, characterized by epithelial‐like morphology, stable growth, and slow proliferation, are well‐suited for long‐term experiments with high reproducibility. In contrast, MKN‐45 cells display poor differentiation, strong invasiveness, and instability, which limit their use in mechanistic studies.^[^ ^28^ ^]^ The MFC line is frequently employed in murine models, providing advantages for in vivo studies of immune mechanisms. The combined use of human MKN‐74 and mouse MFC cells enables xenograft model establishment and facilitates both in vitro and in vivo validation. Therefore, MKN‐74 and MFC were selected for subsequent analyses.

Immunofluorescence staining showed predominant nuclear localization of *SOX9* in MKN‐74 and MFC cells (Figure 3B). To confirm whether SOX9 transcriptionally regulates *TIMP1*, we cloned the *TIMP1* promoter and its mutant variants into the pGL3 luciferase reporter vector (Figure 3C). Dual‐luciferase assays revealed that SOX9 overexpression significantly enhanced luciferase activity in cells transfected with the pGL3‐TIMP1‐WT construct, whereas no effect was observed with the pGL3‐TIMP1‐MUT construct (Figure 3D). Chromatin immunoprecipitation (ChIP) assays further confirmed significant enrichment of *SOX9* at the *TIMP1* promoter region (Figure 3E), demonstrating direct binding and transcriptional activation.

We then manipulated SOX9 expression in MKN‐74 and MFC cells through knockdown and overexpression strategies. The results showed that SOX9 overexpression markedly increased both *SOX9* and *TIMP1* levels, while SOX9 knockdown significantly reduced their expression. Among the shRNA constructs, sh‐SOX9#1 exhibited the strongest inhibitory effect and was selected for subsequent experiments (Figure 3F–I).

These findings demonstrate that the transcription factor SOX9 can activate the expression of TIMP1 in GC cells.

### 2.4 SOX9 Suppression Modulates *TIMP1* Transcription to Inhibit GC Cell Proliferation, Migration, Invasion, and Tumorigenesis

**Figure 4:**
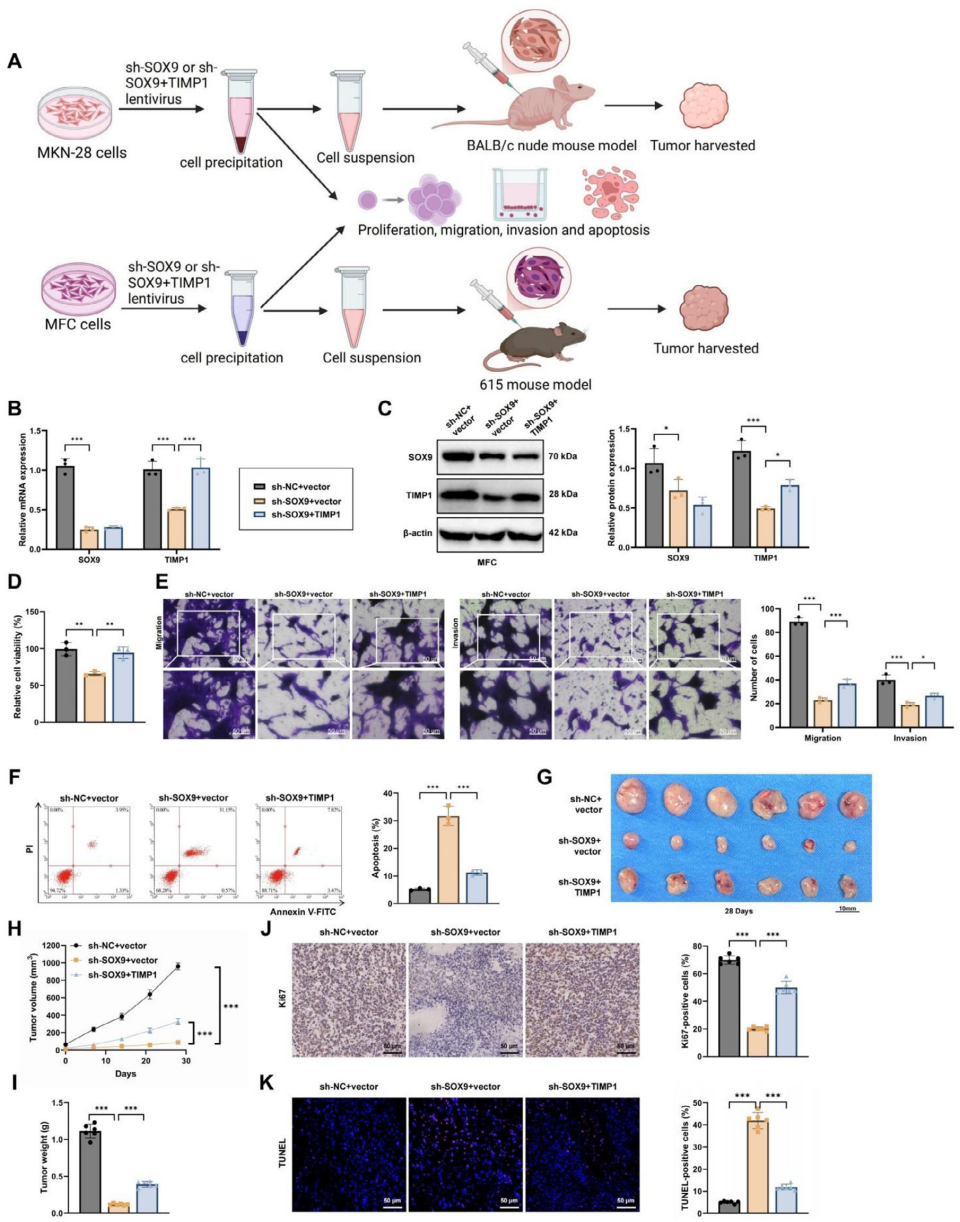
Knockdown of SOX9 regulates *TIMP1* expression, affecting MFC cell proliferation, migration, invasion, apoptosis, and in vivo tumorigenesis. Note: A) Experimental workflow schematic, showing analyses in vitro and a subcutaneous xenograft model in MKN‐74 and MFC cells after knockdown of SOX9 alone or in combination with overexpression of TIMP1; B,C) RT‐qPCR and Western Blot analysis of SOX9 and *TIMP1* mRNA and protein levels in MFC cells after SOX9 knockdown or combined overexpression with TIMP1; D) CCK‐8 assay for cell viability; E) Transwell assay for cell migration and invasion (Scale bars = 50 µm); F) Flow cytometry analysis of cell apoptosis; G) Anatomical diagrams of xenografts from mice 4 weeks post‐transplant; H) Line graph showing changes in xenograft volume in different groups over time; I) Bar graph summarizing xenograft weights 4 weeks post‐transplant in different groups; J,K) Immunohistochemical staining for Ki67 and TUNEL staining assessing cell proliferation and apoptosis in tumor tissues (Scale bars = 50 µm). Data are presented as mean – SD, cell experiments independently repeated three times, animal experiments with six mice per group, ^**^ *p*<0.01, ^***^ *p*<0.001.

Previous studies have indicated that epithelial SOX9 promotes the progression and metastasis of gastric adenocarcinoma by fostering an immunosuppressive TME.^[^ ^25^ ^]^ However, the specific regulatory effect of SOX9 on *TIMP1* transcription and its impact on GC progression remains unclear. We examined the role of the SOX9/*TIMP1* axis in the biological functions of GC cells and their in vivo tumorigenicity (**Figure** **4**A).

Initially, reverse transcription‐quantitative polymerase chain reaction (RT‐qPCR) and Western Blot analyses revealed markedly reduced *SOX9* and *TIMP1* expression in the sh‐SOX9+vector group compared to the sh‐NC+vector group in both MKN‐74 and MFC cell lines. Conversely, *TIMP1* expression was notably increased in the sh‐SOX9+TIMP1 group, while *SOX9* expression remained unchanged (Figure 4B,C; Figure S4A,B, Supporting Information). Functional assays showed that *SOX9* knockdown suppressed cell proliferation, migration, and invasion, whereas these effects were reversed in the sh‐SOX9+TIMP1 group (Figure 4D,E; Figure S4C,D, Supporting Information). Apoptosis assays mirrored these findings, with opposite trends observed in cell proliferation (Figure 4F; Figure S4E, Supporting Information).

In vivo studies using subcutaneous xenograft models with MKN‐74 and MFC cells demonstrated that tumors derived from the sh‐SOX9+vector group were smaller and lighter compared with those from the sh‐NC+vector group. Tumors from the sh‐SOX9+TIMP1 group exhibited increased size and weight (Figure 4G‐I; Figure S4F‐H, Supporting Information). Tumor tissues from the sh‐SOX9+vector group displayed a significant reduction in Ki67‐positive cells and an increase in TUNEL‐positive cells, whereas the sh‐SOX9+TIMP1 group showed the opposite pattern (Figure 4J,K; Figure S4I,J, Supporting Information).

Previous studies have reported that the PI3K/AKT pathway is a major downstream pathway of *TIMP1*, with *TIMP1* activating FAK/PI3K/AKT signaling to promote thyroid cancer progression.^[^ ^29^ ^]^ Consistent with this, analysis of pathway‐related proteins showed that phosphorylation of FAK, PI3K, and AKT was markedly reduced in the sh‐SOX9+vector group but restored in the sh‐SOX9+TIMP1 group (Figure S5A,B, Supporting Information).

Collectively, these results indicate that *SOX9* suppression inhibits GC cell proliferation, migration, invasion, and tumorigenesis by downregulating *TIMP1* transcription and attenuating activation of the FAK/PI3K/AKT signaling pathway.

### 2.5 Knockdown of SOX9 Suppresses *TIMP1* Transcription, Enhancing DC Maturation and Activation

**Figure 5:**
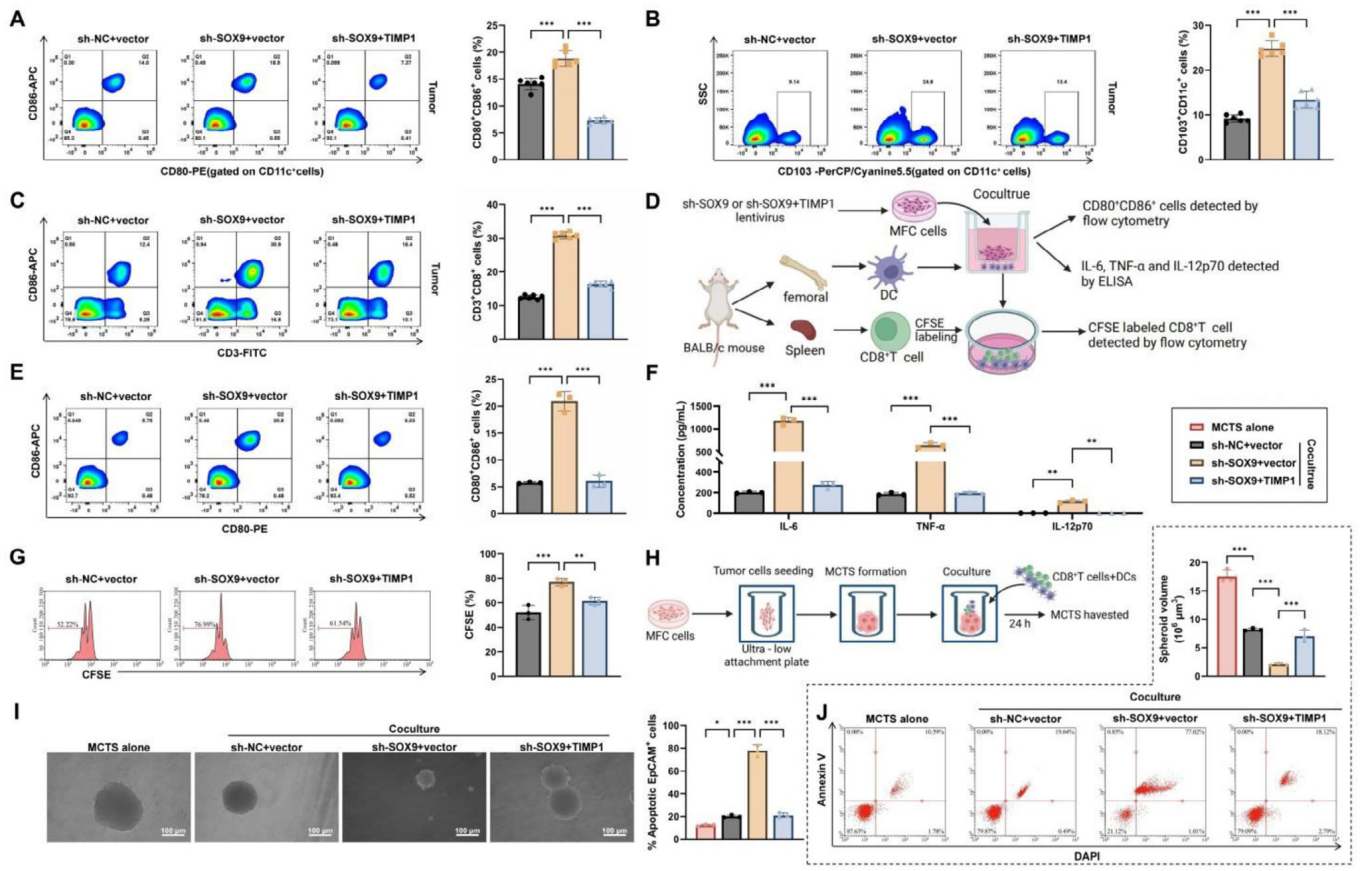
Regulation of DC maturation, activation, and CD8^+^T cell proliferation in GC by SOX9/TIMP1. Note: A–C) Flow cytometry analysis of the proportions of CD80^+^CD86^+^ DCs, CD103^+^ DCs, and CD8^+^T cells in a subcutaneous xenograft model; D) In vitro experimental schematic, illustrating the isolation and culture of DCs from BALB/c mouse bone marrow, co‐cultivation with treated MFC cells, followed by co‐incubation with CD8^+^T cells; E) Flow cytometry analysis of the proportion of CD80^+^CD86^+^ DCs in the in vitro co‐culture system; F) ELISA detection of cytokine levels (IL‐6, TNF‐α, and IL‐12p70) in the in vitro co‐culture system; G) Flow cytometry analysis of the proliferation of CFSE‐labeled CD8^+^T cells; H) Experimental schematic showing the construction of MCTS and the co‐incubation process with DCs and CD8^+^T cells; I) Volume changes of MCTS in each group (Scale bars = 100 µm); J) Flow cytometry analysis of the proportion of apoptotic tumor cells in MCTS. Data presented as mean – SD, cell experiments independently repeated three times, animal experiments with six mice per group, ^*^ *p*<0.05, ^**^ *p*<0.01, ^***^ *p*<0.001.

To determine how SOX9 knockdown influences DC maturation and activation within the TME and its effect on T‐cell priming, subcutaneous MFC tumor‐bearing mice were analyzed. Compared with the sh‐NC+vector group, tumors in the sh‐SOX9+vector group showed significantly higher proportions of CD80⁺, CD86⁺, and CD103⁺ mature DCs, whereas these populations decreased in the sh‐SOX9+TIMP1 group (**Figure** **5**A,B). DCs in TDLNs are crucial for initiating T cell responses to tumor antigens, and similar trends were observed in these cells^[^ ^30^ ^]^ (Figure S6A,B, Supporting Information). Consistent with these findings, CD8⁺ T‐cell infiltration increased in the sh‐SOX9+vector group and decreased in the sh‐SOX9+TIMP1 group within tumor tissues (Figure 5C). These results suggest that SOX9 knockdown promotes DC maturation and activation by inhibiting TIMP1 transcription.

Further in vitro validation was performed (Figure 5D). Bone marrow‐derived dendritic cells (BMDCs) were generated from BALB/c mice and confirmed as CD11c⁺ with high purity (Figure S6C, Supporting Information). CD8^+^T cells were isolated from mouse spleens, yielding 88% purity (Figure S6D, Supporting Information). Co‐culture of BMDCs with MFC cells for 24 h revealed a higher proportion of CD80⁺CD86⁺ DCs in the sh‐SOX9+vector group and a reduced proportion in the sh‐SOX9+TIMP1 group (Figure 5E). Enzyme‐linked immunosorbent assay (ELISA) of culture supernatants confirmed elevated IL‐6, TNF‐α, and IL‐12p70 levels in the sh‐SOX9+vector group, which declined in the sh‐SOX9+TIMP1 group (Figure 5F). Co‐culture with CFSE‐labeled CD8^+^T cells demonstrated enhanced proliferation in the sh‐SOX9+vector group and reduced proliferation in the sh‐SOX9+TIMP1 group (Figure 5G).

MCTS were then used to mimic 3D tissue architecture in vitro.^[^ ^31^ ^]^ MCTS were constructed with MFC cells and co‐cultured with the previously treated DCs and CD8⁺ T cells (Figure 5H). Examination of MCTS integrity showed a significant reduction in volume after co‐culture with DCs and CD8⁺ T cells from the sh‐NC+vector group compared with MCTS alone. The MCTS volume was further reduced in the sh‐SOX9+vector group but increased significantly in the sh‐SOX9+TIMP1 group (Figure 5I). Flow cytometry (FCM) confirmed that spheroid disruption was associated with enhanced apoptosis of tumor cells (Figure 5J).

These findings indicate that *SOX9* knockdown suppresses *TIMP1* transcription, thereby promoting DC maturation and activation, enhancing CD8⁺ T‐cell function, and ultimately inhibiting GC cell growth.

### 2.6 Construction and Characterization of iRGD NPs@si‐SOX9/CL

**Figure 6:**
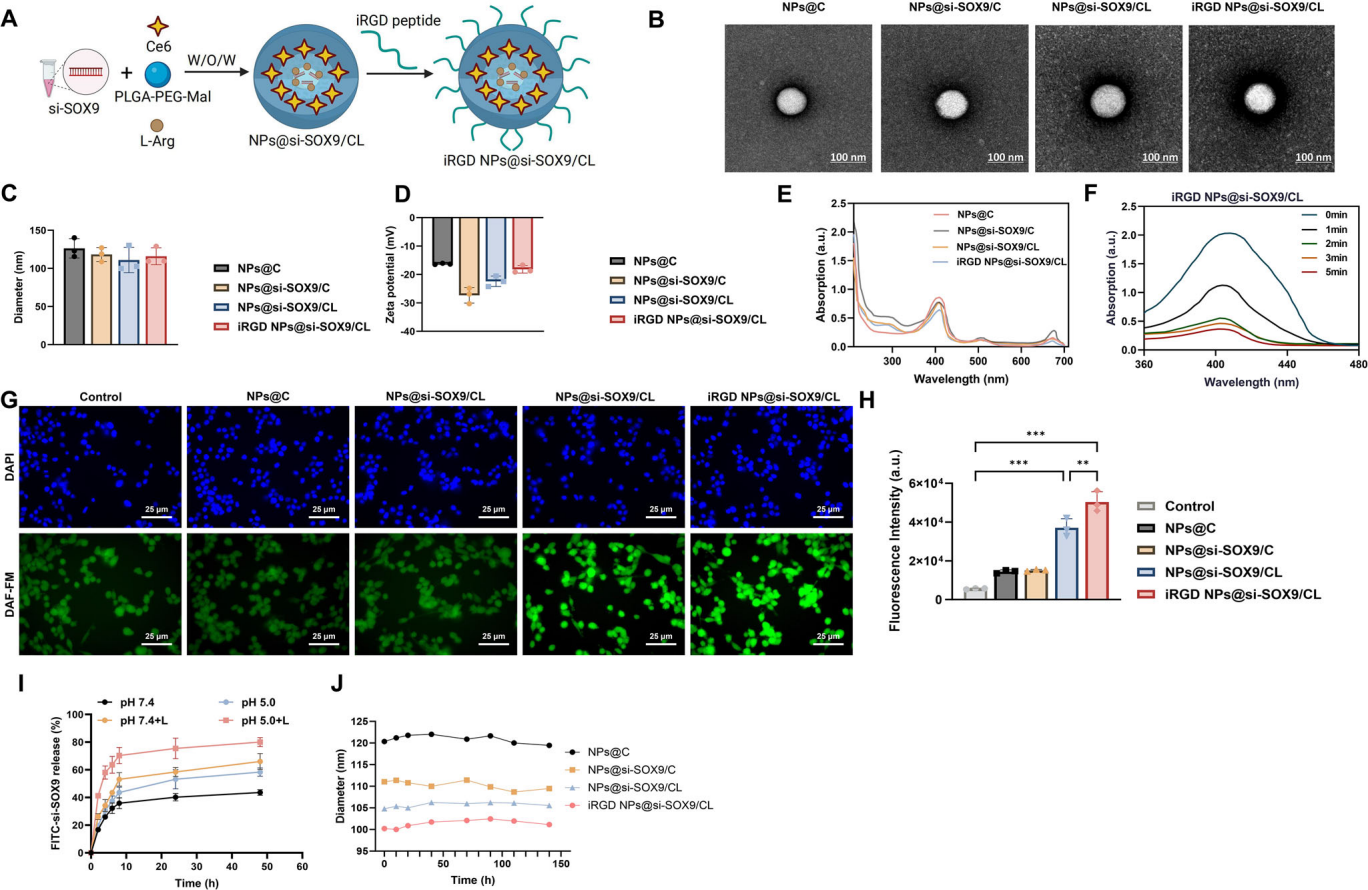
Construction and characterization of iRGD NPs@si‐SOX9/CL. Note: A) Schematic showing the construction process of NPs@si‐SOX9/CL; B) TEM images of NPs@C, NPs@si‐SOX9/C, NPs@si‐SOX9/CL, and iRGD NPs@si‐SOX9/CL (Scale bars = 100 nm); C) DLS analysis of the average diameters of the NPs; D) Zeta potential measurements for each NP formulation; E) UV–vis spectroscopy detecting characteristic absorption peaks of Ce6 in the NPs; F) DPBF assay for the generation of ^1^O_2_ by iRGD NPs@si‐SOX9/CL; G) Fluorescent probe DAF‐FM DA for detecting intracellular NO levels in NP‐treated cell groups; H) Flow cytometry quantitative analysis of intracellular NO levels in NP‐treated cell groups (Scale bars=25 µm); I) FITC‐si‐SOX9 release curves at different pH values (7.4 and 5.0) and before and after NIR irradiation. J) DLS was used to assess the particle size stability of different nanoparticles in serum. Experiments were repeated three times. ^*^ *p*<0.05, ^**^ *p*<0.01, ^***^ *p*<0.001.

To further explore the potential of SOX9 as a target for GC immunotherapy, we developed NPs@si‐SOX9/CL (**Figure** **6**A).

Transmission electron microscopy (TEM) confirmed the spherical structure and uniformity of the NPs (Figure 6B). Dynamic light scattering (DLS) analysis showed that NPs@C, NPs@si‐SOX9/C, NPs@si‐SOX9/CL, and iRGD NPs@si‐SOX9/CL had average diameters of 110–130 nm with zeta potentials ranging from –15.0 to –30.0 mV (Figure 6C–E). We then assessed the effects of different L‐Arg concentrations on ROS and NO production in cells treated with iRGD NPs@si‐SOX9/CL+L. The results indicated that 100 mg mL^−1^ L‐Arg induced the highest ROS and NO levels (Figure S7A, Supporting Information). Optimization experiments revealed that 50 nM siRNA achieved the strongest suppression of SOX9 expression (Figure S7B, Supporting Information). Ultraviolet (UV)–visible–near‐infrared (NIR) spectroscopy of various concentrations of free Ce6 in chloroform identified absorption peaks at ≈500 and 664 nm, from which calibration curves were established (Figure S8A, Supporting Information). In phosphate‐buffered saline (PBS), NPs@C, NPs@si‐SOX9/C, NPs@si‐SOX9/CL, and iRGD NPs@si‐SOX9/CL exhibited characteristic absorption of free Ce6, confirming successful Ce6 encapsulation (Figure 6F). The encapsulation efficiency (EE) and loading content (LC) of Ce6 were 61.2% and 2.3%, respectively. High‐performance liquid chromatography (HPLC) established a calibration curve for free L‐Arg, with an LC of 7.0% (Figure S8B, Supporting Information). Additionally, the siRNA encapsulation efficiencies for NPs@si‐SOX9/C, NPs@si‐SOX9/CL, and iRGD NPs@si‐SOX9/CL were over 80% (Figure S8C, Supporting Information).

To evaluate the protective effect of iRGD NPs@si‐SOX9/CL on siRNA, free si‐RGS19 and nanoparticle‐encapsulated si‐RGS19 were incubated with RNase for different durations. Free si‐RGS19 underwent rapid degradation, whereas si‐RGS19 released from iRGD NPs@si‐SOX9/CL maintained structural integrity for up to four h under RNase exposure (Figure S8D, Supporting Information). The average particle size of iRGD NPs@si‐SOX9/CL remained stable for 24 h in PBS and Dulbecco's modified Eagle medium (DMEM) supplemented with 10% serum (Figure S8E,F, Supporting Information).

Singlet oxygen (^1^O_2_) generation after NIR irradiation of NPs@C, NPs@si‐SOX9/C, NPs@si‐SOX9/CL, and iRGD NPs@si‐SOX9/CL was evaluated using the DPBF assay. Compared with H_2_O, a marked decrease in absorbance was detected, particularly within the first min, indicating rapid ^1^O_2_ production (Figure 6G; Figure S8G, Supporting Information). Intracellular NO levels were assessed using the fluorescent probe 4‐amino‐5‐methylamino‐2′,7′‐dichlorofluorescein diacetate (DAF‐FM DA), which showed green fluorescence in cells exposed to NPs@si‐SOX9/CL and iRGD NPs@si‐SOX9/CL (Figure 6G). FCM confirmed enhanced NO production in both treatment groups (Figure 6H). To examine siRNA release behavior, iRGD NPs@si‐SOX9/CL were incubated in PBS at pH 7.4 and 5.0. Release was markedly accelerated under acidic conditions (pH 5.0) and further enhanced by NIR irradiation (Figure 6I). In addition, nanoparticle stability was verified under serum‐mimicking conditions, showing no detectable aggregation in fetal bovine serum (Figure 6J).

These findings confirm the successful construction of iRGD NPs@si‐SOX9/CL NPs with effective siRNA loading capabilities.

### 2.7 Irgd NPs@si‐SOX9/CL are Taken Up by MFC Cells to Inhibit Growth and Promote DC Maturation

**Figure 7:**
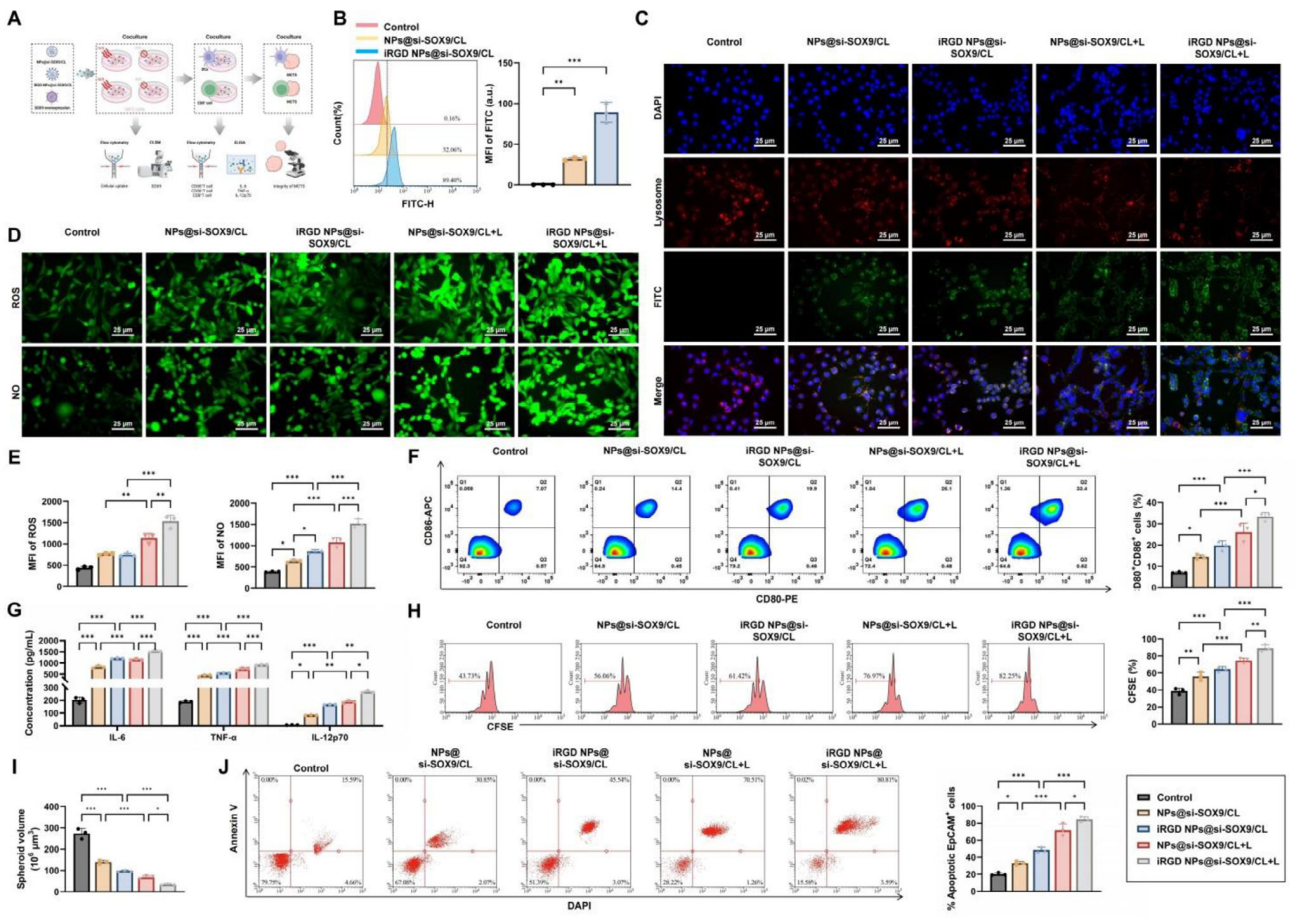
Cellular uptake of iRGD NPs@si‐SOX9/CL and their impact on MFC cell growth and DC maturation. Note: A) Schematic of the in vitro experimental procedure for cellular uptake and co‐culture with iRGD NPs@si‐SOX9/CL; B) Flow cytometry analysis of MFC cell uptake of NPs@si‐SOX9/CL and iRGD NPs@si‐SOX9/CL; C) CLSM imaging of siRNA release from endosomes‐lysosomes in MFC cells treated with NPs@si‐SOX9/CL or iRGD NPs@si‐SOX9/CL, with and without NIR irradiation (Scale bars = 25 µm); D,E) DCFH‐DA and DAF‐FM DA probes assessing the impact of NPs@si‐SOX9/CL or iRGD NPs@si‐SOX9/CL on intracellular ROS and NO generation, with and without NIR irradiation (Scale bars = 25 µm); F) Flow cytometry measurement of CD80^+^CD86^+^ DCs proportion post‐co‐culture with treated MFC cells; G) ELISA analysis of cytokines IL‐6, TNF‐α, and IL‐12p70 in the co‐culture supernatant; H) CFSE labeling to assess the proliferation of CD8^+^T cells co‐cultured with DCs; I) Volume changes of each group's MCTS; J) Flow cytometry analysis of apoptotic tumor cells in MCTS. Data presented as mean – SD, experiments repeated independently three times, ^*^ *p*<0.05, ^**^ *p*<0.01, ^***^ *p*<0.001.

We further explored the in vitro uptake and therapeutic efficacy of iRGD NPs@si‐SOX9/CL (**Figure** **7**A). FCM analysis showed that both NPs@si‐SOX9/CL and iRGD NPs@si‐SOX9/CL were internalized by MFC cells, with uptake of the iRGD‐modified NPs significantly higher than that of non‐modified NPs, confirming that iRGD peptide enhanced si‐SOX9 delivery efficiency (Figure 7B). The uptake of iRGD NPs@si‐SOX9/CL in MCTS was observed. After seven h of incubation, z‐stack confocal laser scanning microscopy (CLSM) detected strong red fluorescence signals at multiple slice depths, indicating efficient penetration and internalization of iRGD NPs@si‐SOX9/CL (Figure S9A, Supporting Information).

CLSM analysis of fluorescein isothiocyanate (FITC)‐labeled si‐SOX9 revealed overlapping green and red fluorescence in cells treated with iRGD NPs@si‐SOX9/CL, suggesting siRNA localization within lysosomes. To investigate endosomal–lysosomal escape, cells were subjected to NIR irradiation. In the iRGD NPs@si‐SOX9/CL+L group, separation of green and red fluorescence was observed, indicating that NIR irradiation facilitated siRNA escape from endosomal–lysosomal compartments. Moreover, these cells displayed more uniform green fluorescence and markedly higher intensity compared with the NPs@si‐SOX9/CL+L group (Figure 7C). Additional CLSM tracking of the escape process confirmed that, prior to irradiation, green signals of aggregated particles fully overlapped with red fluorescence. With prolonged NIR exposure, red fluorescence progressively diminished, while green fluorescence became increasingly diffuse throughout the cytoplasm, demonstrating that NIR/Ce6‐induced ROS disrupted the endosomal membranes (Figure S9B, Supporting Information).

We investigated the expression of SOX9 in cells co‐incubated with iRGD NPs@si‐SOX9/CL with and without NIR irradiation. Both mRNA and protein levels of SOX9 were markedly reduced in the NPs@si‐SOX9/CL and iRGD NPs@si‐SOX9/CL groups relative to controls, with a further decrease observed after NIR irradiation. The iRGD NPs@si‐SOX9/CL+L group exhibited the most pronounced reduction compared with the NPs@si‐SOX9/CL+L group (Figure S9C,D, Supporting Information). To assess oxidative and nitrosative stress, MFC cells were incubated with different NPs for eight h, followed by a 15‐min NIR treatment. Measurement with 2′,7′‐dichlorodihydrofluorescein diacetate (DCFH‐DA) and DAF‐FM DA probes showed significantly higher production of ROS and NO in the nanoparticle‐treated groups. The effect was most evident in the iRGD NPs@si‐SOX9/CL+L group (Figure 7D,E).

Functional assays revealed that cell proliferation, migration, and invasion were significantly inhibited in both NPs@si‐SOX9/CL and iRGD NPs@si‐SOX9/CL groups compared with controls. The inhibitory effect was stronger after NIR irradiation, with iRGD NPs@si‐SOX9/CL+L showing the greatest reduction relative to NPs@si‐SOX9/CL+L (Figure S9E,F, Supporting Information). Conversely, results from apoptosis assays indicated the opposite effect (Figure S9G, Supporting Information). To evaluate biosafety, normal gastric epithelial cells (GES‐1) were co‐incubated with iRGD NPs@si‐SOX9/CL with or without NIR irradiation. Cell viability remained comparable to that of the control group, indicating no cytotoxicity toward normal cells (Figure S9H, Supporting Information).

After co‐incubating MFC cells with DCs for 24 h, FCM revealed a significant increase in CD80⁺CD86⁺ DCs in the NPs@si‐SOX9/CL and iRGD NPs@si‐SOX9/CL groups compared with the control. The increase was further amplified after NIR irradiation, with iRGD NPs@si‐SOX9/CL+L showing the highest proportion of mature DCs (Figure 7F). ELISA results demonstrated elevated secretion of IL‐6, TNF‐α, and IL‐12p70 in the nanoparticle‐treated groups. Cytokine production was further enhanced by NIR irradiation, and the iRGD NPs@si‐SOX9/CL+L group exhibited the strongest effect (Figure 7G). When treated DCs were co‐cultured with CFSE‐labeled CD8⁺ T cells, a significant increase in T‐cell proliferation was observed in the NPs@si‐SOX9/CL and iRGD NPs@si‐SOX9/CL groups relative to the control. Proliferation was further promoted after NIR irradiation, with the iRGD NPs@si‐SOX9/CL+L group showing the greatest enhancement compared with the NPs@si‐SOX9/CL+L group (Figure 7H).

Co‐incubation of MCTS with treated DCs and CD8⁺ T cells resulted in a marked reduction in spheroid volume in the NPs@si‐SOX9/CL and iRGD NPs@si‐SOX9/CL groups compared with the control. The reduction became more pronounced following NIR irradiation, with the iRGD NPs@si‐SOX9/CL+L group exhibiting the strongest suppression relative to the NPs@si‐SOX9/CL+L group (Figure 7I). FCM further confirmed that spheroid disruption was accompanied by enhanced tumor cell apoptosis, indicating that organoid destruction was mediated by active apoptotic processes (Figure 7J).

An additional SOX9 overexpression group (OE‐SOX9) was included for validation. FCM analysis showed a marked reduction in the proportion of CD80⁺CD86⁺ DCs in the OE‐SOX9 group compared with the control, and a significant decrease was also observed in the OE‐SOX9+iRGD NPs@si‐SOX9/CL+L group relative to the iRGD NPs@si‐SOX9/CL+L group (Figure S10A, Supporting Information). Consistently, ELISA results revealed reduced secretion of IL‐6, TNF‐α, and IL‐12p70 in the OE‐SOX9 group compared with the control, and further suppression in the OE‐SOX9+iRGD NPs@si‐SOX9/CL+L group compared with the iRGD NPs@si‐SOX9/CL+L group (Figure S10B, Supporting Information). Co‐culture of treated DCs with CFSE‐labeled CD8⁺ T cells demonstrated that proliferation of CD8⁺ T cells was significantly inhibited in the OE‐SOX9 group compared with the control, and also reduced in the OE‐SOX9+iRGD NPs@si‐SOX9/CL+L group relative to the iRGD NPs@si‐SOX9/CL+L group (Figure S10C, Supporting Information).

When MCTS were co‐cultured with the above‐treated DCs and CD8^+^ T cells, integrity analysis revealed that MCTS volume was significantly increased in the OE‐SOX9 group compared with the control group, and also increased in the OE‐SOX9+iRGD NPs@si‐SOX9/CL+L group compared with the iRGD NPs@si‐SOX9/CL+L group (Figure S10D, Supporting Information). FCM of tumor cell apoptosis further showed that the preservation of MCTS integrity correlated with attenuated apoptosis in tumor cells (Figure S10E, Supporting Information).

These results indicate that iRGD NPs@si‐SOX9/CL are efficiently internalized by MFC cells, thereby inhibiting their growth and promoting the maturation of DCs.

### 2.8 iRGD NPs@si‐SOX9/CL Inhibits GC Cell Tumorigenesis and Promotes DC Activation and Maturation

**Figure 8:**
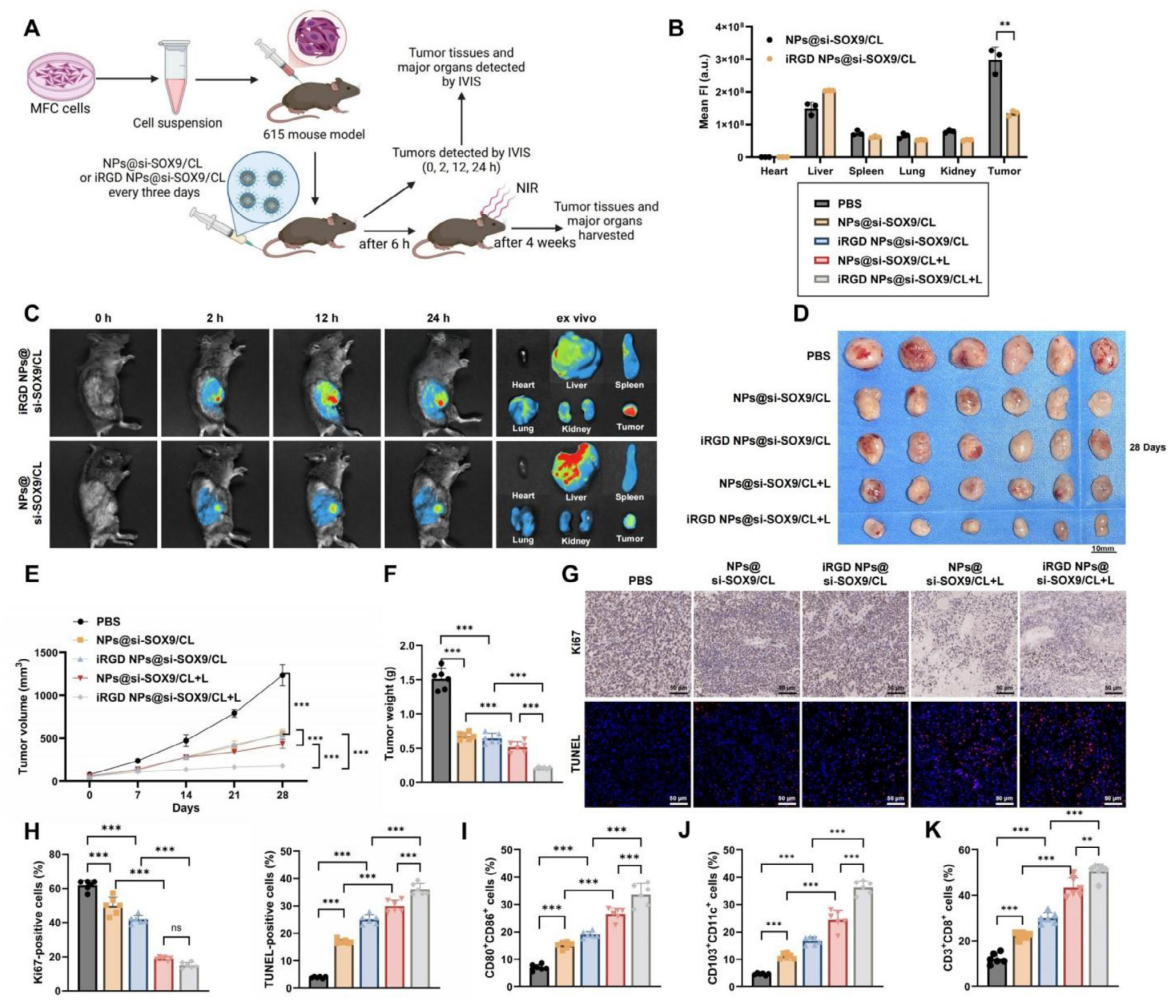
Tumor targeting and antitumor activity of iRGD NPs@si‐SOX9/CL in a subcutaneous xenograft model. Note: A) Experimental procedure schematic; B) Fluorescence imaging of mouse tumor tissues at various time points after intravenous injection of Cy7‐labeled si‐SOX9 NPs@si‐SOX9/CL or iRGD NPs@si‐SOX9/CL; C) Fluorescence imaging and quantitative analysis of main organs and tumor tissues; D–F) Images of subcutaneous tumors in mice, changes in tumor volume over time, and final tumor weights for each treatment group; G,H) Ki67 immunohistochemistry and TUNEL staining to assess proliferative and apoptotic cells in tumor tissues (Scale bars = 50 µm); I,J) Flow cytometry analysis of CD80^+^CD86^+^ and CD103^+^ DCs proportions in tumor tissues of each group; K) Flow cytometry measurement of CD8^+^T cell infiltration in tumor tissues. Data presented as mean – SD, with six mice per group, ^*^ *p*<0.05, ^**^ *p*<0.01, ^***^ *p*<0.001.

The in vivo distribution and therapeutic efficacy of iRGD NPs@si‐SOX9/CL were evaluated using a subcutaneous xenograft model in mice (**Figure** **8**A). We established the model using MFC cells and administered Cy7‐labeled si‐SOX9 via NPs@si‐SOX9/CL or iRGD NPs@si‐SOX9/CL through intravenous injection. Within 24 h, fluorescence imaging showed markedly higher tumor accumulation of iRGD NPs@si‐SOX9/CL compared with NPs@si‐SOX9/CL. Analysis of major organs and tumors further confirmed enhanced enrichment of iRGD‐modified NPs in tumor tissues (Figure 8B,C).

Six h post‐intravenous injection of the NPs, the tumor regions were irradiated with NIR for 20 min. Both tumor weight and volume were significantly reduced in the NPs@si‐SOX9/CL and iRGD NPs@si‐SOX9/CL groups compared with PBS, with more pronounced suppression observed in the NPs@si‐SOX9/CL+L and iRGD NPs@si‐SOX9/CL+L groups. Notably, the iRGD NPs@si‐SOX9/CL+L group exhibited the greatest reduction in tumor burden (Figure 8D–F). Immunohistochemical analysis revealed fewer Ki67‐positive proliferating cells and more TUNEL‐positive apoptotic cells in NP‐treated tumors than in PBS controls, with the strongest effects in the iRGD NPs@si‐SOX9/CL+L group (Figure 8G,H). H&E staining confirmed enhanced tumor suppression following NIR irradiation, while no pathological changes were detected in major organs, demonstrating the biosafety of the NPs in vivo (Figure S11, Supporting Information). These findings suggest that iRGD NPs@si‐SOX9/CL has good biosafety profiles in vivo.

Expression of SOX9/TIMP1/PI3K pathway components in tumor tissues was examined by immunohistochemistry and Western blotting. Compared with PBS controls, both NPs@si‐SOX9/CL and iRGD NPs@si‐SOX9/CL markedly reduced SOX9 and TIMP1 levels, as well as phosphorylation of FAK, PI3K, and AKT. These effects were further amplified under NIR irradiation, with the iRGD NPs@si‐SOX9/CL+L group showing the most pronounced reductions (Figure S12, Supporting Information).

Analysis of subcutaneous MFC xenografts revealed a significant increase in mature and functional DCs (CD80⁺CD86⁺, and CD103⁺). The proportion of mature DCs was further elevated in the NPs@si‐SOX9/CL+L and iRGD NPs@si‐SOX9/CL+L groups compared with their non‐irradiated counterparts, with the highest levels observed in the iRGD NPs@si‐SOX9/CL+L group (Figure 8I,J; Figure S13A,B, Supporting Information). Similar results were observed in the proportion of DCs in TDLNs (Figure S13C,D, Supporting Information). Moreover, CD8⁺ T‐cell infiltration was significantly enhanced in the NPs@si‐SOX9/CL and iRGD NPs@si‐SOX9/CL groups compared with PBS, and further increased in the irradiated groups, with the iRGD NPs@si‐SOX9/CL+L group showing the strongest effect (Figure 8K; Figure S13E, Supporting Information).

These results demonstrate that iRGD NPs@si‐SOX9/CL accumulate efficiently in tumor tissues, inhibit tumor growth by suppressing the SOX9/TIMP1/PI3K axis, and promote DC activation and maturation, thereby achieving potent immunotherapeutic efficacy.

## 3 Discussion

This study elucidates the role of the SOX9/TIMP1/FAK/PI3K signaling axis in shaping the GC microenvironment and demonstrates that iRGD NPs@si‐SOX9/CL combined with PDT enhances the antigen‐presenting ability of DCs and promotes T‐cell activation, thereby improving the GC immune landscape. Previous studies have established the oncogenic function of SOX9 in multiple malignancies, including colorectal, breast, and prostate cancers, where its overexpression drives tumor proliferation, invasion, and therapeutic resistance.^[^ ^32^, ^33^ ^]^ This study is the first to systematically unveil the molecular mechanism by which SOX9 promotes cancer by activating *TIMP1* expression, thereby inhibiting the maturation of DCs and weakening the anti‐tumor immune response. These findings not only broaden the mechanistic understanding of SOX9 in GC progression but also highlight SOX9 as a promising therapeutic target to enhance cancer immunotherapy.

Using scRNA‐seq and ST‐seq, we mapped the cellular interactions within GC tissues and identified significantly enhanced communication between tumor cells and DCs. Spatial transcriptomics revealed distinct expression patterns of SOX9 and TIMP1 within tumor tissues, supporting the conclusion that SOX9 transcriptionally activates TIMP1 to suppress DC maturation. Since DC activation directly determines T cell priming and long‐term immune protection,^[^ ^6^, ^34^, ^35^, ^36^ ^]^ these findings provide mechanistic evidence of how the SOX9/TIMP1 axis mediates GC immune evasion. Previous studies have shown that SOX9 promotes immune evasion by secreting LIF to regulate tumor‐associated macrophage polarization and inhibit CD8^+^ T‐cell function, highlighting its central role in shaping an immunosuppressive microenvironment. Meanwhile, *TIMP1*, as an extracellular matrix regulator, has been implicated in immunosuppression and poor prognosis in the TME.^[^ ^25^, ^37^ ^]^ Our study is the first to systematically demonstrate that SOX9 functions as a key transcription factor activating *TIMP1* expression, thereby inhibiting DC maturation and directly impairing their antigen presentation and immune‐activating capacities. This elucidates, at the molecular level, an important mechanism underlying immune evasion in GC.

In recent years, nanotechnology has made remarkable progress in medicine, particularly in tumor drug delivery and immunotherapy.^[^ ^38^, ^39^ ^]^ Here, we designed PLGA NPs encapsulating si‐SOX9 and Ce6, further modified with iRGD peptides to enhance tumor targeting. Compared with conventional drug delivery methods, this nanocarrier system exhibits superior targeting ability and biocompatibility, enabling significant drug accumulation at tumor sites. The iRGD NPs@si‐SOX9/CL system efficiently delivers si‐SOX9 to suppress SOX9 expression, downregulates its downstream target *TIMP1*, and consequently blocks the FAK/PI3K/AKT signaling pathway. This markedly enhances DC maturation and antigen presentation. Previous studies indicated that the SOX9/TIMP1 axis suppresses DC costimulatory molecule (CD80, CD86) expression, leading to immune evasion and impaired T‐cell activation. Our findings show that, in combination with Ce6‐mediated PDT, this nanosystem further promotes tumor apoptosis and antigen release. Restoration of DC function and activation of CD8^+^ T cells synergistically strengthen antitumor immunity, thereby remodeling the immune microenvironment. This is consistent with recent reports highlighting the critical role of DC activation in improving responses to immunotherapy and PDT.^[^ ^8^, ^40^ ^]^ Thus, nanomedicine‐based PDT targeting the SOX9/TIMP1/FAK/PI3K axis provides a novel strategy for precision immunotherapy in GC.

PDT is an important noninvasive cancer treatment that employs photosensitizers under specific light irradiation to generate ROS, thereby inducing tumor apoptosis.^[^ ^41^, ^42^ ^]^ Selective accumulation of photosensitizers enhances tumor cell sensitivity to light, enabling precise tumor ablation.^[^ ^43^, ^44^ ^]^ However, PDT still faces clinical challenges such as tumor hypoxia and limited photosensitizer delivery efficiency.^[^ ^45^, ^46^ ^]^ To address this, we incorporated L‐Arg as an NO donor to improve oxygen availability in the TME, thereby enhancing PDT efficacy. L‐Arg releases NO in response to elevated H_2_O_2_ in tumors, increasing oxygen content, boosting ROS production, and elevating oxidative stress during PDT. Traditional PDT is limited by insufficient photosensitizer targeting, poor tissue penetration, and tumor immune evasion. Recent studies indicate that combining siRNA delivery systems with PDT can overcome these limitations.^[^ ^11^ ^]^ On the one hand, nanocarriers (e.g., iRGD‐modified NPs) efficiently deliver siRNAs (e.g., si‐SOX9) to silence key immune evasion pathways such as SOX9/TIMP1/FAK/PI3K, thereby releasing the blockade on DC maturation and enhancing immune activation. On the other hand, PDT‐induced tumor antigen release further amplifies immune responses. Together, these synergistic effects significantly remodel the tumor immune microenvironment and enhance antitumor responses. Therefore, the integration of PDT with siRNA nanodelivery not only improves targeting and efficacy but also reverses immune evasion, offering a promising new approach for solid tumor therapy.^[^ ^16^ ^]^ The mechanism and therapeutic efficacy of the innovative nano‐delivery strategy iRGD NPs@si‐SOX9/CL combined with PDT have been supported by theoretical and experimental evidence across multiple research fields. Our study confirmed that SOX9 enhanced TIMP1 expression, activated the FAK/PI3K signaling cascade, and inhibited DC maturation, thereby suppressing antigen presentation and immune activation. The combination of nanotechnology‐based PDT with siRNA‐mediated silencing of immune‐evasion molecules such as SOX9 enhances photosensitizer delivery efficiency and remodels the immune microenvironment, restoring DC and CD8^+^ T‐cell function and substantially improving tumor‐specific immune responses. From a safety perspective, this nanosystem selectively accumulates in tumors with minimal off‐target toxicity, exhibiting excellent biocompatibility.^[^ ^15^ ^]^ With its precision targeting, low toxicity, and multi‐modal immune activation, this strategy offers an innovative and safe immunotherapeutic approach for solid tumors.

Compared with conventional GC therapies such as surgery, chemotherapy, targeted therapy, or single‐agent immunotherapy, our iRGD NPs@si‐SOX9/CL platform integrates siRNA nanodelivery and PDT to specifically suppress the SOX9/TIMP1/FAK/PI3K axis while simultaneously enhancing DC function. This effectively reverses immune evasion and strengthens CD8^+^ T‐cell antitumor activity. Compared with traditional PDT, this nanosystem demonstrates superior tumor targeting, higher accumulation and release efficiency of photosensitizers and siRNA, and enhanced immune activation and antitumor efficacy. In vivo, it showed greater tumor suppression and immune activation than monotherapies.^[^ ^47^ ^]^ Moreover, emerging evidence indicates that combined nanotherapies may overcome chemotherapy resistance and the challenge of “immune‐cold” tumors.^[^ ^48^, ^49^, ^50^, ^51^ ^]^ Advantages include precise delivery, multi‐pathway regulation, and favorable safety, whereas potential limitations, such as long‐term in vivo stability, safety, and clinical reproducibility, warrant large‐scale and long‐term validation.^[^ ^52^ ^]^ Collectively, this nanosystem provides a multi‐targeted, immune‐enhancing strategy for GC with unique advantages and broad clinical prospects.

In addition, compared with conventional PD‐1 blockade or SOX9 small‐molecule inhibitors, the iRGD‐modified nanocarrier system shows remarkable superiority. Unlike PD‐1 antibodies, which act only on T‐cell immune checkpoints, iRGD NPs@si‐SOX9/CL silence the SOX9/TIMP1/FAK/PI3K axis, relieve immunosuppression, and simultaneously induce immunogenic tumor cell death, activating both DCs and CD8^+^ T cells for dual immune regulation.^[^ ^53^, ^54^ ^]^ Compared with small‐molecule inhibitors, siRNA nanodelivery offers potent and specific gene silencing with higher efficiency and selectivity. Moreover, iRGD modification greatly improves tumor accumulation and release while reducing uptake by normal tissues, thereby increasing safety.^[^ ^55^ ^]^ Taken together, iRGD NPs@si‐SOX9/CL outperforms traditional therapeutic strategies in targeting, efficacy, and immune activation, offering a novel multi‐modal precision therapy for GC.

Despite these promising findings, several limitations should be acknowledged. Although our nanoparticle‐based strategy demonstrated robust efficacy in murine models, the greater heterogeneity and complexity of human tumors may yield different therapeutic outcomes. Validation in larger‐scale cohorts and clinical trials will be essential to confirm these findings. Further optimization of nanoparticle design for diverse TMEs should also be pursued. In addition, exploration of potential synergistic effects with other immunomodulatory agents represents an important direction for future research.

## 4 Conclusion

This study utilized scRNA‐seq and ST‐seq to elucidate the pivotal role of the SOX9/TIMP1 axis in immune evasion in GC and confirmed the mechanism by which iRGD NPs@si‐SOX9/CL enhance DC function and promote anti‐tumor immune responses during PDT by inhibiting the SOX9/TIMP1/FAK/PI3K signaling axis (Graphical abstract). Experimental results demonstrated that NPs@si‐SOX9/CL significantly inhibited GC cell proliferation, migration, and invasion, while simultaneously promoting DC maturation and CD8^+^T cell activation, effectively improving the GC immune microenvironment. These findings provide a promising targeted therapeutic strategy for GC. This study innovatively applied scRNA‐seq and ST‐seq to analyze the GC immune microenvironment, integrating PDT combined with siRNA NP delivery to address the limitations of traditional therapies in targeting and immune evasion. The study elucidates the molecular mechanism by which SOX9 activates TIMP1 to inhibit DC maturation and proposes a nano‐immunotherapy strategy with substantial scientific value and clinical potential for GC. With continued advances in nanotechnology, PDT, and tumor immunology, this strategy holds promise for delivering more precise and effective personalized treatment options for patients with GC.

## 5 Experimental Section

### Data Acquisition and Processing

scRNA‐seq, ST‐seq, and bulk transcriptomic datasets of GC were obtained from the GEO (http://www.ncbi.nlm.nih.gov/geo/) and TCGA databases. scRNA‐seq data were subjected to quality control, normalization, dimensionality reduction, and clustering using standard pipelines. Major cell populations were annotated based on canonical markers and public reference databases. ST‐seq data were integrated with scRNA‐seq profiles to achieve spatial deconvolution and visualization of cellular distributions within GC tissues.

### Bioinformatic and Functional Analyses

Bulk RNA‐seq data from TCGA‐STAD were analyzed to identify DEGs between tumor and normal tissues. PPI networks and KEGG pathway enrichment analyses were conducted to characterize the functional roles of candidate genes. To further prioritize key targets, LASSO regression, survival analysis, and transcription factor binding prediction were performed. Immune cell infiltration was assessed using established deconvolution algorithms, and correlations between gene expression and immune infiltration were systematically evaluated. Detailed protocols, including dataset accession numbers, parameter thresholds, and software versions, are provided in the Supporting Information.

### Cell Culture

Human GC cell lines HGC‐27 (CC‐Y1228; RRID: CVCL_1276) and MKN‐45 (CC‐Y1358; RRID: CVCL_0434), mouse GC cell line MFC (CC‐Y2059; RRID: CVCL_8723), and human normal gastric mucosal cell line GES‐1 (CC‐Y1572; RRID: CVCL_8156) were purchased from Shanghai Enzyme‐linked Biotechnology Co., Ltd. (Shanghai, China), while the MKN‐74 cell line (CL‐0730; RRID: CVCL_2791) was obtained from Procell (Wuhan, Hubei, China). The HEK‐293T cell line (CRL‐3216; RRID: CVCL_0063) was purchased from ATCC (Manassas, VA, USA). All lines were authenticated by STR profiling and confirmed free of mycoplasma contamination. MKN‐74, MKN‐45, and MFC cells were maintained in RPMI 1640 medium (Thermo Fisher Scientific, USA) supplemented with 10% fetal bovine serum (FBS, Thermo Fisher Scientific, USA) and 1% penicillin–streptomycin. HGC‐27, GES‐1, and 293T cells were cultured in DMEM (Thermo Fisher Scientific, USA) containing 10% FBS and 1% antibiotics. Cells were incubated at 37 °C in 5% CO_2_, with medium refreshed every 24 h and passaged every 72 h using 0.25% trypsin.

### Lentiviral Transduction and Cell Grouping

SOX9 shRNA lentiviral plasmids (sh‐SOX9) were constructed using the lentiviral silencing vector pGLVU6/Puro (C06002, GenePharma, Shanghai, China). Packaging plasmids pCMV‐dR8.91 (Fenghui Biotech, China) and pMD2.G (Addgene, USA) were co‐transfected into HEK‐293T cells at a 3:3:1 ratio using the calcium phosphate method. The knockdown sequences used were as follows: Human: sh‐SOX9‐1 (TRCN0000020385; target sequence: ACCTTCGATGTCAACGAGTTT), sh‐SOX9‐2 (TRCN0000020386; target sequence: CTCCACCTTCACCTACATGAA). Mouse: sh‐SOX9‐1 (TRCN0000086165; target sequence: CAGACTCACATCTCTCCTAAT), sh‐SOX9‐2 (TRCN0000086167; target sequence: CTCCACCTTCACTTACATGAA), and sh‐NC (CTCGCTTGGGCGAGAGTAA).

At 24 h post‐transfection, the medium was replaced with serum‐free medium for 24–30 h. Viral supernatants were harvested, filtered through 0.45 µm membranes, and concentrated by ultracentrifugation at 80,000 g for 2 h at 4 °C. Viral titers were determined with a p24 ELISA kit(L00938, Jiangsu, China). MFC and MKN‐74 cells were infected with virus‐containing supernatant, and stable knockdown lines were established following puromycin selection (4 µg mL^−1^ for 14 days, reduced to 2 µg mL^−1^ after 9 days). SOX9 overexpression constructs were generated using the LV4 lentiviral vector (GenePharma, Shanghai, China), with an empty vector as a control.

The experimental groups are as follows: (1) Vector and SOX9 groups; sh‐NC, sh‐SOX9#1 (sh‐SOX9), and sh‐SOX9#2 groups; (2) sh‐NC+vector, sh‐SOX9+vector, and sh‐SOX9+TIMP1 groups. (3) Control, NPs@si‐SOX9/CL, iRGD NPs@si‐SOX9/CL, NPs@si‐SOX9/CL+L, and iRGD NPs@si‐SOX9/CL+L groups. For NPs@si‐SOX9/CL and NPs (si‐SOX9), siRNA concentration was 50 nm, applied for 24 h. L refers to NIR irradiation (670 nm, 0.1 W cm^−2^) for 5 min. (4) Control, OE‐SOX9, iRGD NPs@si‐SOX9/CL+L, and OE‐SOX9+iRGD NPs@si‐SOX9/CL+L. For iRGD NPs@si‐SOX9/CL, the siRNA concentration was 50 nm, applied for 24 h. “*L*” refers to NIR irradiation (670 nm, 0.1 W cm^−2^) for 5 min. Cells from each group were collected for subsequent analysis.

### Cellular Functional Assays

Cell proliferation was assessed using the CCK‐8 assay, while cell migration and invasion were evaluated through Transwell assays with or without Matrigel coating. FCM was employed to detect apoptosis in gastric cancer cell lines and to analyze DC maturation and CD8^+^ T‐cell activation from tumor tissues and tumor‐draining lymph nodes. In addition, apoptosis in MCTS was analyzed following dissociation into single‐cell suspensions. Detailed gating strategies are shown in Figure S14 (Supporting Information). Additionally, detailed experimental procedures, including cell culture conditions, reagent information, and staining protocols, are provided in the Supporting Information.

### Immunofluorescence Staining for SOX9 Localization

MKN‐74 and MFC cells were seeded onto sterile glass slides, fixed with 4% paraformaldehyde (PFA; I28800, Thermo Fisher Scientific, USA), permeabilized with 0.1% Triton X‐100, and blocked with 1% bovine serum albumin (BSA). Slides were incubated overnight at 4 °C with rabbit anti‐SOX9 antibody (1:100, Abcam), followed by Cy3‐conjugated goat anti‐rabbit secondary antibody (1:1000, Abcam) for 60 min at room temperature. Nuclei were counterstained with DAPI (Beyotime, China) and mounted with antifade medium (Vector Laboratories, USA). Fluorescence images were acquired using a CLSM (LSM 510 META, Carl Zeiss AG).

### ChIP Assay

ChIP assays were performed with a commercial kit (Cat#26157, Thermo Fisher Scientific, USA). MKN‐74 cells were crosslinked with 1% formaldehyde for 10 min, quenched with 0.125 M glycine, washed, and harvested. Cells were lysed to obtain nuclei, which were further disrupted in SDS lysis buffer containing protease inhibitors. Chromatin was sheared by sonication into 200–1000 bp fragments. Immunoprecipitation was carried out overnight at 4 °C using anti‐SOX9 (1:50, ab185230, Abcam, UK) and anti‐IgG (1:50, ab171870, Abcam, UK) antibodies, with IgG serving as the negative control. SOX9‐bound chromatin was captured using Pierce Protein A/G magnetic beads, followed by centrifugation at 12 000 × g for 5 min. After extensive washing to remove nonspecific complexes, crosslinking was reversed at 65 °C overnight. The recovered DNA was purified and used as a template for quantitative PCR to assess *TIMP1* promoter enrichment. The primer sequences were: 5’‐TTGGGGGATGTGGGTGATTG‐3’ and 5’‐ACTATTCCTTCCAAGCCGCC‐3’.

### Luciferase Reporter Assay

PCR was used to amplify the *TIMP1* promoter region containing SOX9 binding sites, which was subsequently cloned into the pGL3‐Promoter Vector (E1761, Promega, Madison) to generate the pGL3‐TIMP1 plasmid. A mutant version of the binding site was created and cloned into the same vector to produce the pGL3‐TIMP1‐MUT plasmid. 293T cells were seeded in six‐well plates at 2 × 10^5^ cells per well. Once the cells adhered, Lipofectamine 3000 reagent (L3000150, Invitrogen, Thermo Fisher Scientific, Waltham, MA, USA) was used to co‐transfect pGL3‐TIMP1‐WT or pGL3‐TIMP1‐MUT with SOX9, a negative control (vector), and the Renilla luciferase plasmid pRL‐TK (E2241, Promega, Madison) into the 293T cells. After 48 h, dual‐luciferase activity was quantified using the Dual‐Luciferase Reporter Assay Kit (11420ES, YEASEN, Shanghai, China). Firefly luciferase activity was normalized to Renilla activity, and signals were recorded with a Glomax 20/20 luminometer (Promega).

### Activation and Maturation of DCs In Vitro

BMDCs were isolated from the femurs of BALB/c mice (211, Vital River, Beijing, China) and cultured in RPMI 1640 medium supplemented with 10% FBS, 1% antibiotics, IL‐4 (10 ng mL^−1^, PeproTech, AF‐214‐14), and GM‐CSF (20 ng mL^−1^, PeproTech, AF‐315‐03). After seven days, CD11c^+^ DCs were generated, and purity was verified by FCM.

MFC cells from each treatment group were seeded in the upper chamber of a Transwell system (0.4 µm pore size, Corning, USA) and incubated for 24 h. The treated MFC cells were then co‐cultured with CD11c^+^ DCs and seeded in the lower chamber at a 2:1 ratio for 24 h. CD11c^+^ DCs were collected and stained with the following antibodies: FITC‐conjugated anti‐CD11c (117306, Biolegend, USA), APC‐conjugated anti‐CD86 (159215, Biolegend, USA), and PE‐conjugated anti‐CD80 (104707, Biolegend, USA). The stained cells were analyzed by FCM.

### Isolation and Proliferation Assay of CD8^+^T Cells

Spleens from BALB/c mice were collected in cold PBS containing 2% FBS, mechanically dissociated through a 40 µm strainer using a 10 mL syringe plunger, and centrifuged at 300 g for 10 min. The resulting splenocytes were resuspended in cryopreservation medium (RPMI 1640 with 65% FBS and 10% DMSO, 1 × 10^8^ cells mL^−1^) and stored at −80 °C until use. For co‐culture experiments, frozen splenocytes were rapidly thawed at 37 °C, resuspended in recovery medium (RPMI 1640 supplemented with 10% FBS and 1% antibiotics), and centrifuged at 270 g for 5 min. CD8⁺ T cells were isolated using the EasySep Mouse CD8⁺ T Cell Enrichment Kit (Stem Cell Technologies, #19853), and purity was confirmed by FCM. Isolated CD8⁺ T cells were stimulated overnight with anti‐CD3 antibody (2 µg mL^−1^, A19017, ABclonal, China), followed by 48 h stimulation with anti‐CD28 antibody (2 µg mL^−1^, A20346, ABclonal, China) and recombinant IL‐2 (100 U mL^−1^, RP01384, ABclonal, China). Cells were cultured in RPMI 1640 supplemented with 10% FBS, 1% antibiotics, 10 mm HEPES, 2 mm L‐glutamine, 1 mM sodium pyruvate, MEM non‐essential amino acids, and 55 µm β‐mercaptoethanol. Medium was replaced every 2–3 days to maintain a minimum density of 2 × 10^6^ cells mL^−1^. Cells were counted on day seven and maintained under identical conditions until day 14.

For proliferation assays, CD8⁺ T cells were labeled with CFSE (2.5 µm, 65‐0850‐84, Thermo Fisher Scientific, USA) at 37 °C for 15 min, followed by quenching with serum‐containing medium for 5 min. After three PBS washes, CFSE‐labeled CD8⁺ T cells were co‐cultured with CD11c⁺ DCs (from MFC co‐culture) at a 5:1 ratio for 48 h. Non‐adherent cells were collected and analyzed by FCM to assess CFSE dilution as a measure of T cell proliferation.

### Cytokine Level Detection

After co‐culturing the MFC cells from each treatment group with DCs for 24 h, the culture supernatants were collected, and the levels of IL‐6, TNF‐α, and IL‐12p70 were measured using ELISA kits (88‐7064‐88/88‐7324‐88/88‐7121‐88, Invitrogen, Thermo Fisher Scientific, USA).

### Generation and Co‐Culture of MCTS

MCTS were generated by seeding 10^4^ MFC cells in a 96‐well ultra‐low attachment plate (Costar, 7007, Corning, USA) and culturing in a complete RPMI 1640 medium. After 5 days of incubation, MCTS were formed and maintained at 37 °C in a CO_2_ incubator (5% CO_2_). The culture medium was replaced every two days.

For co‐culture, suspended or loosely adherent DCs and CD8⁺ T cells were collected by centrifugation, while adherent DCs were detached with cell dissociation buffer (1 mL/well, 13151‐014, Thermo Fisher Scientific, USA). When MCTS reached ≈3 × 10^4^ cells, a mixture of 3 × 10^5^ DCs and CD8⁺ T cells was added per well. Co‐culture was continued for 24 h before harvesting spheroids for downstream analysis.

### MCTS Volume Calculation

After co‐culturing MCTS with DCs and CD8^+^T cells, the spheroids were seeded into 96‐well plates and imaged using an inverted microscope at magnifications of 2x to 4x. Images were analyzed with Icy software to measure spheroid length (L) and width (W). Volumes were calculated using the formula: V = (L × W × W) / 2

### Preparation of si‐SOX9, Ce6, and L‐Arg‐Loaded NPs

PLGA NPs loaded with si‐SOX9, the photosensitizer Ce6, and L‐Arg (NPs@si‐SOX9/CL) were synthesized using a double‐emulsion method (W/O/W). Briefly, 50 mg PLGA‐PEG‐Mal (20–5.0 kDa, LA: GA = 50:50; HY‐148776, MCE, USA) and 2 mg Ce6 (HY‐13594, MCE, USA) were dissolved in 2 mL chloroform (PHR1552, Sigma–Aldrich, USA). Subsequently, 400 µL of deionized water containing 40 mg L‐Arg (100 mg mL^−1^, HY‐N0455, MCE, USA; mass ratio L‐Arg: Ce = 20:1,^[^ ^12^ ^]^) was added. Separately, 800 ng si‐SOX9 was dissolved in 100 µL RNase‐free water (9012, Takara, Japan) and incorporated to generate the primary O/W emulsion using a probe sonicator (TL‐ST150, Tenlin, China). For the secondary emulsification, 8 mL of 4% (w/v) PVA solution (363170, Sigma–Aldrich, USA) was added, and the W/O/W double emulsion was formed by sonication. The mixture was stirred for 2 h to evaporate chloroform, followed by centrifugation with Milli‐Q water to remove unencapsulated materials. The final NPs@si‐SOX9/CL were collected by centrifugation (10 000 rpm, 8 min). NPs containing varying L‐Arg concentrations (25, 50, and 200 mg mL^−1^) and siRNA concentrations (12.5, 25, and 100 nm under 100 mg mL^−1^ L‐Arg) were also prepared.

Next, the NPs@si‐SOX9/CL NPs were conjugated with iRGD peptide (HY‐P0122, MCE, USA) by the thiol groups on the Mal moiety of Mal–PEG–PLGA and the thiol groups on iRGD peptide, and incubated for 24 h at a 4:1 molar ratio of NPs to iRGD. Cysteine quenched the unreacted Mal groups on the NPs (Cys; HY‐Y0337, MCE, USA). The resulting solution was transferred to an ultrafiltration device (MWCO 100 kDa; Millipore Corporation, Bedford, MA) and centrifuged to form the targeted nanoprobe, iRGD NPs@si‐SOX9/CL. The NPs were dried overnight and stored under 2‐8 °C, protected from light and oxygen, in PBS buffer.^[^ ^56^ ^]^

The siRNA duplex targeting SOX9 was designed online and synthesized by GenePharma (Shanghai, China). The 21‐nt sequences were: sense, 5′‐AUUACAAAGUCCAAACAGGCA‐3′; antisense, 5′‐CCUGUUUGGACUUUGUAAUUA‐3′.

### Characterization of iRGD NPs@si‐SOX9/CL

The size, particle size distribution, and ζ‐potential of iRGD NPs@si‐SOX9/CL were measured at 25 °C using a Malvern Zetasizer (Nano ZS90, Malvern Instruments, UK). For morphological observation, 10 µL of the sample was placed on a carbon‐coated copper grid (300 mesh), incubated for 3 min, negatively stained with 1% uranyl acetate for 15 s, air‐dried, and imaged using a TEM (TECNAI‐10, Philips, The Netherlands) at 120 kV. Fluorescence spectra of NPs@si‐SOX9/CL, NPs@si‐SOX9/C, and NPs@C were recorded with a Fluoromax‐4 fluorometer (Horiba Jobin Yvon Inc.) using 600 nm excitation.

Encapsulation efficiency (EE%) of si‐SOX9 was quantified using the RiboGreen assay. Briefly, 10 µL of siRNA‐loaded NPs (iRGD NPs@si‐SOX9/CL or NPs@si‐SOX9/CL) was mixed with 117 µL TE buffer (A) or 2% Triton X‐100 (B), vortexed, and incubated with diluted RiboGreen reagent (1:200) for 5 min. Fluorescence intensity was measured at 480/520 nm on a microplate reader, and EE% was calculated as: EE% = (Fluorescence of B‐Fluorescence of A) / Fluorescence of B × 100%

L‐Arginine content was determined by demulsifying NPs with DMSO followed by HPLC analysis (Shimadzu LC‐20AD, Japan). Chromatographic conditions were: 4.6 × 150 mm, 5 µm column; mobile phase phosphate buffer/acetonitrile (81:19, v/v); detection at 210 nm; flow rate 1.0 mL min^−1^. LC and EE of L‐Arg and Ce6 were calculated as:

(1) $$EE_{\mathrm{Ce}6}=\left(\mathrm{Weight}\;\mathrm{of}\;\mathrm{Ce}6\;\mathrm{loaded}\;\mathrm{in}\;\mathrm{NPs}/\mathrm{Total}\;\mathrm{weight}\;\mathrm{of}\;\mathrm{Ce}6\right)\times 100\%$$

(2) $$\begin{array}{ccc} & & LC_{L-\mathrm{Arg}}{/}_{\mathrm{Ce}6}\\ & & \;=\left(\mathrm{Weight}\;\mathrm{of}L-\mathrm{Arg}\;\mathrm{or}\;\mathrm{Ce}6\;\mathrm{loaded}\;\mathrm{in}\;\mathrm{NPs}/\mathrm{Total}\;\mathrm{weight}\;\mathrm{of}\;\mathrm{NPs}\right)\\ & & \;\times 100\% \end{array}$$

### Serum Stability

To evaluate in vitro stability, NPs were incubated in 10% FBS (Atlanta Biologicals, GA, USA) at 37 °C for 5 days. Particle size was measured by DLS every six h within the first 12 h, and then every 24 h up to five days.^[^ ^57^ ^]^

### Si‐SOX9 Release Kinetics

To evaluate release behavior, FITC‐labeled iRGD NPs@si‐SOX9/CL (HY‐66019, MCE, USA) were suspended in PBS and loaded into Float‐A‐Lyzer dialysis bags (MWCO 300 kDa). Dialysis was performed at 37 °C under pH 7.4 and pH 5.0 conditions. At predetermined time points (2, 4, 6, 8, 24, and 48 h), aliquots of the external PBS solution were collected and replaced with equal volumes of fresh PBS. Released siRNA was quantified by fluorescence measurement (excitation/emission: 495/519 nm) using a standard curve of siRNA concentration vsfluorescence intensity.

### Stability of iRGD NPs@si‐SOX9/CL

The RNase resistance of free si‐SOX9 and iRGD NPs@si‐SOX9/CL was evaluated by agarose gel electrophoresis. Samples were incubated with RNase ONE™ (10 µg mL^−1^; M4261, Promega, USA) at 37 °C for 0, 15, 30, 60, 120, and 240 min. NPs were collected by centrifugation (12,000 rpm, 10 min), dissolved in chloroform, and siRNA was extracted with 0.5 M NaCl containing 0.1% SDS. Extracted siRNA was analyzed on 3% agarose gels stained with GelRed and visualized under UV illumination.

To evaluate the stability of iRGD NPs@si‐SOX9/CL, the NPs were incubated in PBS buffer and cell culture medium containing 10% FBS for 0, 3, 6, 12, and 24 h. The NP solutions were analyzed for size by DLS at each time point.

### In Vitro Cellular Uptake and Lysosomal Escape of iRGD NPs@si‐SOX9/CL in MFC Cells

MFC cells (1 × 10^5^/well) were seeded in 12‐well plates and cultured overnight, then incubated with FITC‐labeled NPs@si‐SOX9/CL or iRGD NPs@si‐SOX9/CL for two h. After PBS washing, cells were collected, resuspended in PBS, and analyzed by FCM. For intracellular distribution, MFC cells (1 × 10^4^/well) were plated in 35 mm confocal dishes and treated with FITC‐si‐SOX9, NPs@si‐SOX9/CL, or iRGD NPs@si‐SOX9/CL for 2 h. Nuclei and lysosomes were stained with DAPI and LysoTracker Red DND‐99 (L7528, Invitrogen, USA), and images were acquired using CLSM.

To evaluate lysosomal escape, cells were incubated with NPs for 2 h, stained as above, and subjected to NIR irradiation (670 nm, 0.1 W cm^−^^2^). Fluorescence redistribution was recorded by CLSM and quantified by FCM at 0, 2, 4, 6, and 8 min post‐irradiation.

### Detection of ^1^O_2_ Generation

^1^O_2_ production was measured using 1,3‐diphenylisobenzofuran (DPBF, 105481, Sigma–Aldrich, USA) as a probe. Briefly, NPs were dispersed in H_2_O and mixed with 10 µg mL^−1^ DPBF/DMSO, including groups containing NPs@C (5 µg mL^−1^ Ce6), NPs@si‐SOX9/C, NPs@si‐SOX9/CL, and iRGD NPs@si‐SOX9/CL. Samples were exposed to 670 nm laser irradiation (0.1 W cm^−^^2^, 5 min), and the absorbance at 416 nm was monitored using a UV–vis spectrophotometer to quantify DPBF degradation.

### Measurement of ROS Production Using DCFH‐DA Probe

The production of ROS in MFC cells was assessed using the fluorescent probe DCFH‐DA (excitation/emission = 488 nm/530 nm). MFC cells (1 × 10^5^ cells per well) were seeded in 6‐well plates and incubated for 24 h, followed by treatment with PBS (control), NPs@si‐SOX9/CL, or iRGD NPs@si‐SOX9/CL (Ce6, 10 µg mL^−1^) for 24 h, with or without liquid paraffin overlay. NIR‐treated groups were irradiated at 670 nm (0.1 W cm^−^^2^, 5 min). Cells were fixed in 4% PFA for 20 min, counterstained with DAPI for 15 min, and imaged by CLSM to visualize ROS production.

### Measurement of NO Production Using DAF‐FM DA Probe

The generation of NO was measured using the sensitive fluorescent probe DAF‐FM DA (λex/λem = 495 nm/515 nm). MFC cells (1 × 10^5^ cells per well) were seeded in 6‐well plates for 24 h, then incubated with PBS, NPs@C, NPs@si‐SOX9/C, NPs@si‐SOX9/CL, or iRGD NPs@si‐SOX9/CL (Ce6, 10 µg mL^−1^) for an additional 24 h. After treatment, NIR‐irradiated groups were exposed to a 670 nm laser (0.1 W cm^−^^2^, 5 min). Cells were washed, fixed with 4% formaldehyde, stained with DAPI for 15 min, and visualized under a fluorescence microscope.

### CLSM Measurement of MCTS Penetration

MCTS with a diameter of ≈700 µm were cultured in fresh RPMI 1640 medium containing iRGD NPs@si‐SOX9/CL (Ce6: 10 µg mL^−1^). The MCTS were incubated with iRGD NPs@si‐SOX9/CL at 37 °C in the dark for seven h. After incubation, the MCTS were washed three times with a medium. CLSM images were captured using Z‐stack CLSM to assess NP penetration.

### In Vivo Biodistribution of NPs

Male 615 mice (4–5 weeks old, 18–22 g; Beijing HFK Bioscience Co., Ltd., 13004A)^[^ ^58^ ^]^ were maintained under SPF conditions with controlled temperature (25–27 °C), humidity (45–50%), and a 12 h light/dark cycle. Mice were acclimated for 1 week prior to experimentation and fasted for 12 h before administration, with free access to water. All animal procedures were approved by the Institutional Animal Care and Use Committee of First Hospital of China Medical University (No. CMUXN2023081).

Subcutaneous xenograft tumors were established by injection of MFC cells. Tumor‐bearing mice were randomized into two groups (n = 3) and intravenously injected with 200 µL Cy7‐labeled NPs@si‐SOX9/CL or iRGD NPs@si‐SOX9/CL (2 mg kg^−1^ Cy7‐siRNA). Whole‐body fluorescence imaging was conducted using the IVIS‐200 system (PerkinElmer, Waltham, MA, USA) at 0, 2, 12, and 24 h post‐injection. After 24 h, tumors and major organs (heart, liver, spleen, lungs, kidneys) were excised for ex vivo imaging. Fluorescence intensity was quantified using ImageJ software to evaluate NP biodistribution.^[^ ^59^, ^60^ ^]^

### In Vivo Antitumor Study

Male BALB/c nude mice (4–5 weeks old, weighing 18–22 g) were purchased from Vital River (401, Beijing, China). Animals were used for in vivo studies and were cared for in accordance with the Guide for the Care and Use of Laboratory Animals. All animal procedures were approved by the Institutional Animal Care and Use Committee of First Hospital of China Medical University (No. CMUXN2023081).

Stable transfected MFC cells (1 × 10^6^ cells) were subcutaneously injected into the backs of 615 mice, or MKN‐74 cells (1 × 10^6^ cells) were injected into the backs of nude mice to establish subcutaneous xenograft tumor models. Eighteen mice of each strain were randomly assigned to three groups (n = 6 per group): sh‐NC + vector, sh‐SOX9 + vector, and sh‐SOX9 + TIMP1. Tumor width (W) and length (L) were measured weekly using calipers, and tumor volume (V) was calculated as V = (W^2^ × L) / 2. Four weeks post‐injection, mice were euthanized, and tumors were excised, photographed, and weighed.^[^ ^61^, ^62^ ^]^ Tumor tissues were divided into two portions: one was fixed in 4% PFA for immunohistochemical staining, and the other was frozen in liquid nitrogen and stored at −80 °C for subsequent analysis.

MFC cells (1 × 10^6^) were injected subcutaneously into the backs of 615 mice to establish a subcutaneous xenograft tumor model. The mice were randomly divided into five groups (n = 6 per group): (1) PBS group, (2) NPs@si‐SOX9/CL group, (3) iRGD NPs@si‐SOX9/CL group, (4) NPs@si‐SOX9/CL + L group, and (5) iRGD NPs@si‐SOX9/CL + L group. After successful tumor establishment, the mice were administered NPs@si‐SOX9/CL or iRGD NPs@si‐SOX9/CL via tail vein injection every three days, with si‐SOX9 and Ce6 dosed at 2 and 1 mg kg^−1^, respectively. For the NIR irradiation groups (670 nm, 0.1 W cm^−2^), a 20‐min NIR exposure was applied six h after NP administration.^[^ ^63^ ^]^

Four weeks after treatment, tumors and major organs (heart, liver, spleen, lungs, kidneys) were harvested for analysis. Organs were processed for histopathological evaluation by H&E staining. Briefly, tissues were fixed in 4% PFA, dehydrated through graded ethanol, cleared in xylene, embedded in paraffin, sectioned, and mounted. Sections were stained with hematoxylin (5 min), differentiated in 1% hydrochloric acid ethanol, counterstained with eosin (2 min), dehydrated, and mounted. Pathological alterations were examined under a light microscope.^[^ ^60^, ^64^ ^]^

### Immunohistochemical Staining

Immunohistochemical (IHC) staining was performed on xenograft tumor sections to evaluate the expression of Ki67, SOX9, and TIMP1. Protein expression was semi‐quantitatively evaluated based on staining intensity and the percentage of positive cells. Detailed antibody information and scoring criteria are provided in the Supporting Information.

### TUNEL Staining

Apoptotic cells in tumor sections were detected using the TUNEL assay. Nuclei were counterstained with DAPI, and the apoptotic index was calculated as the percentage of TUNEL‐positive cells among total nuclei. Detailed experimental procedures are available in the Supporting Information.

### RT‐qPCR

Total RNA from tissues and cells was extracted using Trizol reagent (15596026, Invitrogen, Thermo Fisher Scientific). The RNA concentration and purity were measured using a Nanodrop2000 spectrophotometer (1011U, Nanodrop, Thermo Fisher Scientific, USA). According to the manufacturer's protocol, RNA was reverse‐transcribed into cDNA using the PrimeScript RT Reagent Kit (RR047A, Takara, Japan). RT‐qPCR was performed using the Fast SYBR Green PCR Kit (RR820A, Takara, Japan) on an ABI PRISM 7300 RT‐PCR system (Applied Biosystems). β‐actin was used as the reference gene, and gene expression was calculated using the 2^−ΔΔCt^ method. The experiment was repeated three times. Primer sequences are shown in Table S2 (Supporting Information).

### Western Blot

Cells and tissues were lysed on ice for 30 min in RIPA buffer containing PMSF (1%, P0013B, Beyotime, Shanghai, China). Lysates were centrifuged at 14,000 g for 20 min at 4 °C, and supernatants were collected. Protein concentrations were quantified by the BCA assay. Samples were mixed with 5× loading buffer, boiled at 100 °C for 10 min, and 50 µg of protein was separated by SDS‐PAGE and transferred to PVDF membranes (FFP28, Beyotime, China). Membranes were blocked with 5% non‐fat milk for 1 h at room temperature and incubated overnight at 4 °C with the following primary antibodies: anti‐SOX9 (rabbit, 1:1000, #82630, CST, USA), anti‐TIMP1 (mouse, 1:1000, sc‐21734, Santa Cruz, USA), anti‐FAK (rabbit, 1:1000, 3283, CST, USA), anti‐p‐FAK (rabbit, 1:1000, 3285, CST, USA), anti‐PI3K (rabbit, 1:1000, ab191606, Abcam, UK), anti‐p‐PI3K (rabbit, 1:1000, ab278545, Abcam, UK), anti‐AKT (rabbit, 1:1000, 4691, CST, USA), anti‐p‐AKT (rabbit, 1:2000, 4060, CST, USA), and anti‐β‐actin (rabbit, 1:1000, ab8226, Abcam, UK). β‐actin served as a loading control. After washing with PBST, membranes were incubated with HRP‐conjugated goat anti‐rabbit or goat anti‐mouse IgG secondary antibodies (1:10,000, BA1054/BA1050, BOSTER, Wuhan, China) for 1 h at room temperature. Protein bands were visualized with ECL reagent (AR1172, BOSTER, China) and imaged using an Amersham Imager 600 system (USA). Densitometric quantification was performed with ImageJ software. All experiments were repeated three times.

### Statistical Analysis

All experiments were performed at least in triplicate, and data wereexpressed as the mean – standard deviation (SD). Comparisons between two groups were conducted using independent samples t‐tests. For comparisons among three or more groups, one‐way ANOVA followed by Tukey's HSD post hoc test was applied. For non‐normally distributed data or data with unequal variances, the Mann–Whitney U test or Kruskal–Wallis H test was used. Statistical analyses were performed using GraphPad Prism 9.5 (GraphPad Software, Inc.) and R software. A two‐tailed p < 0.05 was considered statistically significant.

### Ethical Statement

All animal experiments were approved by the Animal Ethics Committee of First Hospital of China Medical University. Animals were used for in vivo studies and were cared for in accordance with the Guide for the Care and Use of Laboratory Animals.

## Conflict of Interest

The authors declare no conflict of interest.

## Author Contributions

B.Z., Y.Z., and H.W. These authors were regarded as co‐first authors B.Z., Y.Z., and H.W. designed and performed the experiments, analyzed data, and drafted the manuscript. L.G. conceived and supervised the project, provided critical revisions, and secured funding. All authors discussed the results and approved the final version of the manuscript.

## Supporting information



Supporting Information

Supporting Information

Supporting Information

## Data Availability

The data that support the findings of this study are available from the corresponding author upon reasonable request.
