# Supplementary material for: Targeting the SOX9/TIMP1 Axis with iRGD‐Conjugated Nanoplatform Enhances Dendritic Cell Function and Photodynamic Immunotherapy in Gastric Cancer
Source: Adv Sci (Weinh). 2025 Nov 21;13(3):e10500. doi: 10.1002/advs.202510500 (PMC12806509; doi:10.1002/advs.202510500)
Supplement: Supplementary file 1 — Supporting Information [file ADVS-13-e10500-s001.docx]

1. **Materials and Methods**
   1. **Download and Analysis of scRNA-seq Data from GC Patient Tumor Samples**

The scRNA-seq data for GC tissue samples were downloaded from the GEO database ([http://www.ncbi.nlm.nih.gov/geo/](http://www.ncbi.nlm.nih.gov/geo/)), including datasets GSE163558 and GSE184198. The GSE184198 dataset contains scRNA-seq data for one primary GC tissue sample and one adjacent normal tissue sample. From the GSE163558 dataset, we selected data from three primary GC samples (GSM5004180, GSM5004181, GSM5004182) and one adjacent normal tissue sample (GSM5004183). The data from these six samples (four primary GC tissues and two adjacent normal tissues) were analyzed using the "Seurat" package in R. Quality control was performed with the criteria of 200 < nFeature_RNA < 5000 and percent.mt < 20, and the top 2000 most variable genes were selected for further analysis.

- 1. **UMAP Clustering, Cell Annotation, and Cell-Cell Communication Analysis**

To reduce the dimensionality of the scRNA-seq dataset, PCA was performed on the top 2000 highly variable genes. The ElbowPlot function in Seurat was used to select the first 30 principal components (PCs) for downstream analysis. The "FindClusters" function in Seurat identified the major cell subpopulations, with a resolution set to res = 0.4. Non-linear dimensionality reduction was then performed using the UMAP algorithm. Cells were annotated based on known cell lineage-specific marker genes and by cross-referencing with the CellMarker online database. Cell-cell communication was analyzed using the "CellChat" package in R. DEGs in epithelial cells between the two sample groups were identified using the "Limma" package in R, with the criteria |log_2_FC| > 1 and adj*p*.value < 0.05.

- 1. **Download and Analysis of ST-seq Data from GC Patient Tumor Samples**

The ST-seq dataset GSE203612 was downloaded from the GEO database. We selected two primary GC tissue samples (GIST1: GSM6177607 and GIST2: GSM6177609) from the GSE235672 dataset and their corresponding ST sequencing data. The scRNA-seq data were integrated with the 10x Visium spatial transcriptomics data from the GSE203612 dataset using Seurat's anchor-based integration pipeline. This integration allowed the transfer of cell type annotations from scRNA-seq data to spatial transcriptomics data. The cell type predictions from Seurat were then loaded into the "SPOTlight" R package, which was used to annotate and visualize cell types at each spatial location.

- 1. **Download and Analysis of Transcriptomic Data**

Transcriptomic expression data for GC (TCGA-STAD) were downloaded from the TCGA database. The dataset includes 375 GC tissue samples and 32 adjacent normal tissue samples. Differential expression analysis was performed using the R package edgeR (version 3.34.0), applying the criteria |log_2_FC| > 1 and FDR < 0.05 to identify DEGs between the tumor and control groups. Specifically, genes with expression values of zero in more than 50% of the samples were removed. The DGEList function was used to construct the input matrix, followed by data normalization using the calcNormFactors function, and differential expression analysis with the exactTest function. The results were visualized using volcano plots and violin plots of gene expression differences generated with the ggplot2 package in R.

- 1. **PPI Network and KEGG Enrichment Analysis**

Genes associated with "gastric cancer" were retrieved from the GeneCards database ([https://www.genecards.org/](https://www.genecards.org/)) with a score ≥20 as the filtering criterion. The DEGs from both scRNA-seq and TCGA-STAD datasets were intersected with disease-related genes, and the PPI network for these intersecting genes was constructed using the STRING database ([https://string-db.org/](https://string-db.org/)). The PPI network was visualized using Cytoscape software. KEGG pathway enrichment analysis was performed using the clusterProfiler package, and significantly enriched pathways were selected based on *p*-value. The results were visualized in a bubble plot.

- 1. **Target Gene Screening**

LASSO is a regression method used for variable selection and regularization to improve the accuracy and interpretability of predictive models. LASSO regression analysis used the R package glmnet to identify disease-related target genes further. Survival analysis was performed using the Kaplan-Meier Plotter website, with a total sample size of n = 875. Potential transcription factor binding sites for target genes were predicted using the JASPAR database. Immune cell infiltration in tumor and adjacent normal tissues from the TCGA-STAD dataset was analyzed using the CIBERSORT algorithm. Spearman correlation analysis was then performed to assess the relationship between the expression of key genes and immune cell infiltration. The correlation was visualized through heatmaps and scatter plots.

- 1. **Cell Counting Kit-8 (CCK-8) Assay for Cell Proliferation**

MKN-74, GES-1, and MFC cells, in good growth condition, were seeded at a density of 6 × 10^3^ cells per well in a 96-well plate. After overnight incubation, cells were treated according to the experimental groups. Then, 10 μL of CCK-8 solution (96992, Sigma-Aldrich, USA) was added to each well. After 1 hour of incubation at 37 °C in a humidified incubator, the absorbance at 450 nm was measured using an Epoch Microplate Spectrophotometer (Bio-Tek, Winooski, VT, USA). Other treatment groups were also analyzed for cell viability using the CCK-8 assay. Each group included six replicates, and the experiment was repeated three times.

- 1. **Transwell Assay**

MKN-74 and MFC cells were treated under various conditions and subjected to Transwell invasion assays. 50 μL of Matrigel (356234, Corning, USA) was used to coat the upper chamber of the Transwell, which was incubated at 37 °C for 30 minutes to allow gel solidification. After washing the coated chambers with serum-free medium, cells were resuspended at a concentration of 2.5 × 10^4^ cells/mL. 100 μL of the cell suspension was added to the upper chamber, and 500 μL of medium containing 10% FBS was added to the lower chamber. After 24 hours, the chambers were removed, and cells in the upper chamber were wiped off with a cotton swab. The chambers were then fixed with 4% paraformaldehyde (PFA) at room temperature for 30 minutes. Cells were stained with 0.1% crystal violet for 30 minutes. Five random areas were selected, and images were captured using an inverted microscope (IXplore Pro, Olympus, Japan) to count the number of cells. In the migration assay, Matrigel was omitted. The experiment was repeated three times.

- 1. **Flow Cytometry**

Cell Apoptosis Detection: Apoptosis in MKN-74 and MFC cells was assessed using the Flow Cytometry Apoptosis Kit (APOAF, Sigma Aldrich). A cell suspension (1×10^5^/mL) was prepared, and 5 µL of FITC-Annexin V and PI were sequentially added. The cells were incubated in the dark at room temperature for 20 minutes. Apoptosis was then measured using a Guava® easyCyte™ 6-2L flow cytometer, and data were analyzed using CellQuest Pro software. Apoptotic cells were identified in the second and third quadrants.

Analysis of DCs and CD8^+^T Cells: Mouse tumor tissue and tumor-draining lymph nodes (inguinal lymph nodes, TDLNs) were cut into small pieces and digested for 30 minutes in medium containing 100 units/mL collagenase IV and 50 µg/mL DNase I. The resulting cell suspension was filtered through a 70 μm filter, and red blood cells were lysed using ACK buffer before cell counting. Cells were incubated in fluorescence-activated cell sorting buffer containing 0.5% bovine serum albumin (BSA) and were stained with APC-anti-CD86, PE-anti-CD80, FITC-anti-CD11C, PE/Cyanine5-anti-CD103, FITC-anti-CD3, APC-anti-CD8a, and PerCP/Cyanine5.5-anti-CD8a antibodies. CD80^+^CD86^+^ and CD103^+^ DCs as well as CD8^+^T cells were detected using a FACSAria II flow cytometer, and data were analyzed with FlowJo software.

MCTS Tumor Cell Apoptosis Analysis: multicellular tumor spheroids (MCTS) were dissociated into single-cell suspensions using trypsin digestion. Tumor cells were labeled with APC-anti-CD326 antibody, followed by co-staining with FITC-Annexin V and DAPI. Apoptosis analysis was then conducted using flow cytometry. Detailed gating strategies are shown in Figure S14.

- 1. **Immunohistochemical Staining**

The xenograft tumor tissues were fixed in 4% PFA for one week. The samples were then embedded in paraffin and sectioned to 4 μm thickness. Antigen retrieval was performed by heating the sections in 0.1 M citrate buffer (pH 6.0) in a microwave at 100 °C for 10 minutes. The slides were incubated overnight at 4 °C with primary antibodies: rabbit anti-Ki67 (1:200, ab16667, Abcam, UK), rabbit anti-SOX9 (1:100, #82630, CST, USA), and mouse anti-TIMP1 (1:100, sc-21734, Santa Cruz, USA). Subsequently, the slides were incubated for 1 hour at room temperature with HRP-conjugated goat anti-rabbit or anti-mouse secondary antibodies (1:1000, ab6721/ab205719, Abcam, UK). The immunoreaction was visualized using 0.05% diaminobenzidine (DAB) with 0.01% hydrogen peroxide (DA1010, Solarbio, Beijing, China). The slides were then counterstained with hematoxylin for 5 minutes and washed with tap water. The sections were differentiated in 1% hydrochloric acid ethanol for 4 seconds and blued with tap water for 20 minutes. The presence of brown-yellow staining identified positive protein cells. The immunostaining intensity was graded as follows: negative = 0, weak = 1, moderate = 2, strong = 3. The percentage of positively stained cells was calculated, and the final score was determined by multiplying the intensity score by the percentage of positive cells (score = intensity × percentage of positive cells). Images were captured using a Nikon ECLIPSE Ti microscope system (Fukasawa, Japan) and processed with Nikon software.

- 1. **TUNEL Staining**

The samples from each group were fixed with 4% PFA at room temperature for 15 minutes, followed by permeabilization with 0.25% Triton X-100 for 20 minutes. The cells were then incubated with 5% BSA (36101ES25, YEASEN, Shanghai, China) and stained using the TUNEL reagent (C1089, Beyotime, Shanghai). Afterward, the sections were counterstained with DAPI staining solution in the dark. Apoptotic cells were imaged using a confocal microscope. TUNEL-positive cells (red fluorescence) indicate apoptotic cells, while DAPI-stained nuclei (blue fluorescence) represent total cells. The apoptosis rate was calculated by selecting five fields per group and determining the ratio of apoptotic cells to total cells: apoptosis rate = (number of apoptotic cells / total number of cells) × 100%.
