# Supplementary material for: Targeting the SOX9/TIMP1 Axis with iRGD‐Conjugated Nanoplatform Enhances Dendritic Cell Function and Photodynamic Immunotherapy in Gastric Cancer
Source: Adv Sci (Weinh). 2025 Nov 21;13(3):e10500. doi: 10.1002/advs.202510500 (PMC12806509; doi:10.1002/advs.202510500)
Supplement: Supplementary file 2 — Supporting Information [file ADVS-13-e10500-s002.docx]

**
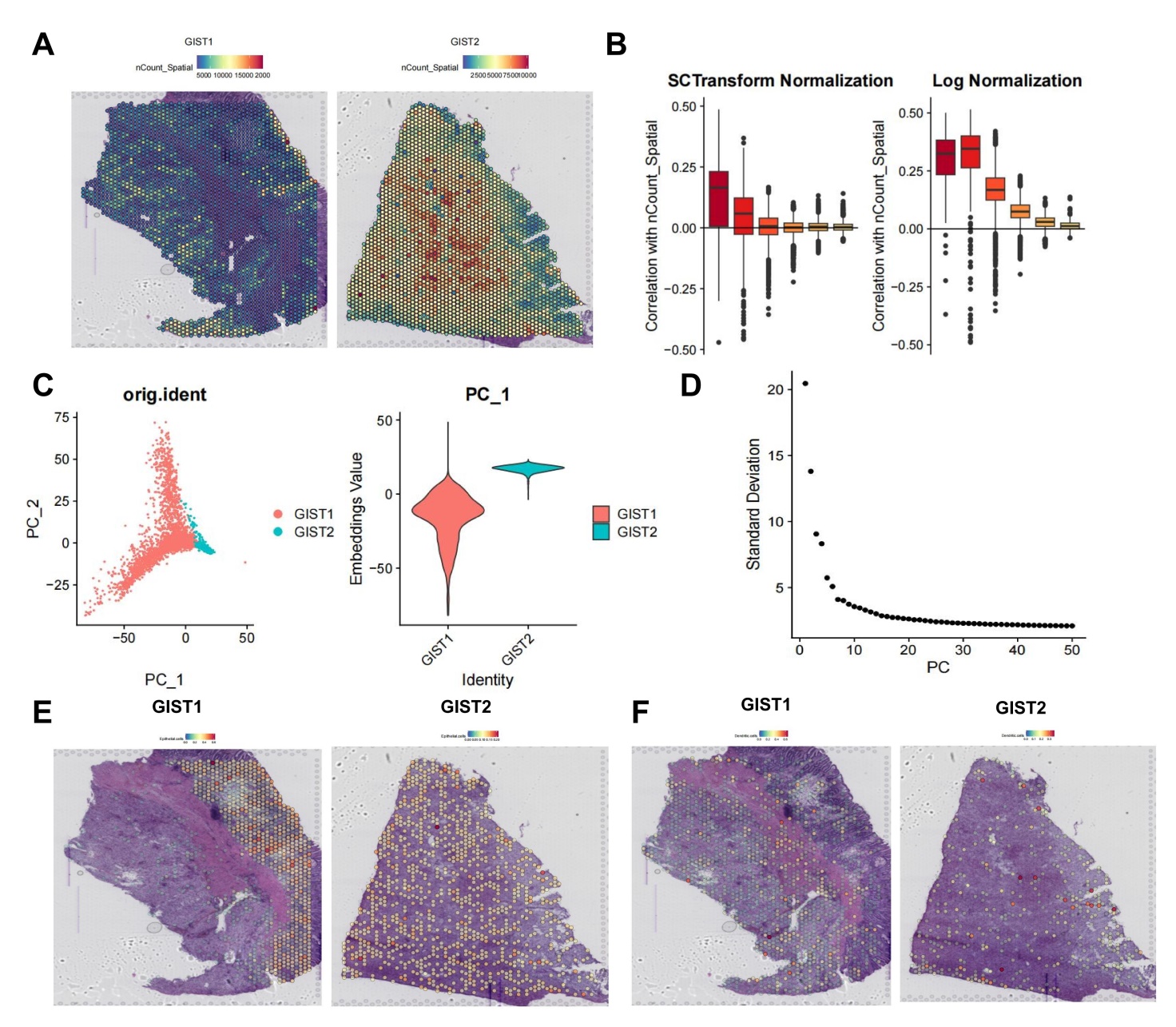
**

**Figure S1. Standardization and PCA dimension reduction of ST data.**

Note: (A) Distribution of nCount_Spatial across tissue sections in ST data, with redder colors indicating higher expression of nCount_Spatial at that spot; (B) Results of SCTransform and LogNormalize standardization of ST data; (C) PCA analysis showing the distribution of cells along PC_1 and PC_2, each point representing a cell; (D) Distribution of SDs across PCs, with important PCs exhibiting larger SDs; (E-F) Distribution of epithelial cells (E) and DCs (F) on GC tissue sections. Sample size n=2.

**
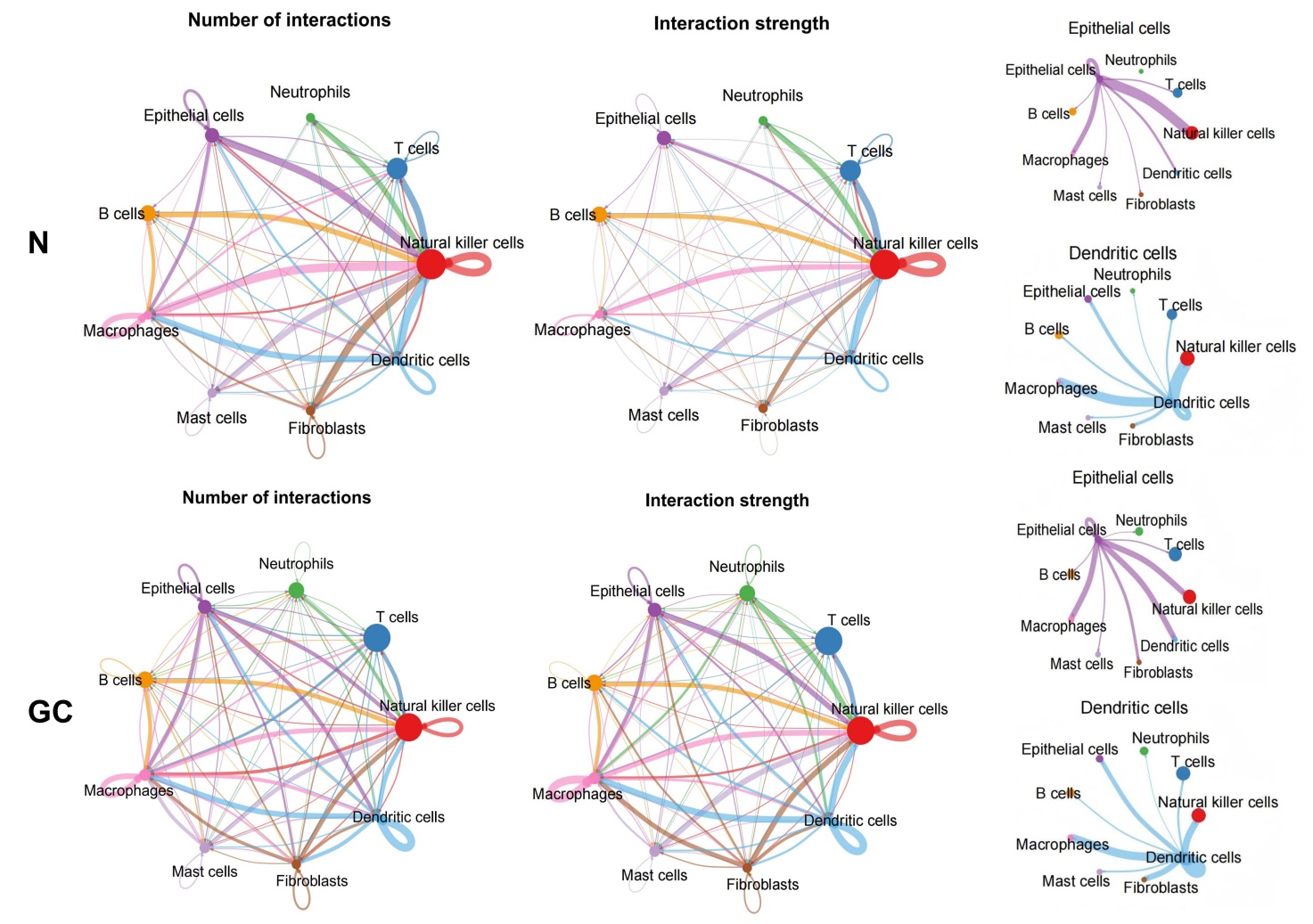
**

**Figure S2. Communication analysis between cells in scRNA-seq data.**

Note: Line thickness represents the number of pathways or interaction strength, N for adjacent normal tissue, GC for GC tissue.

**
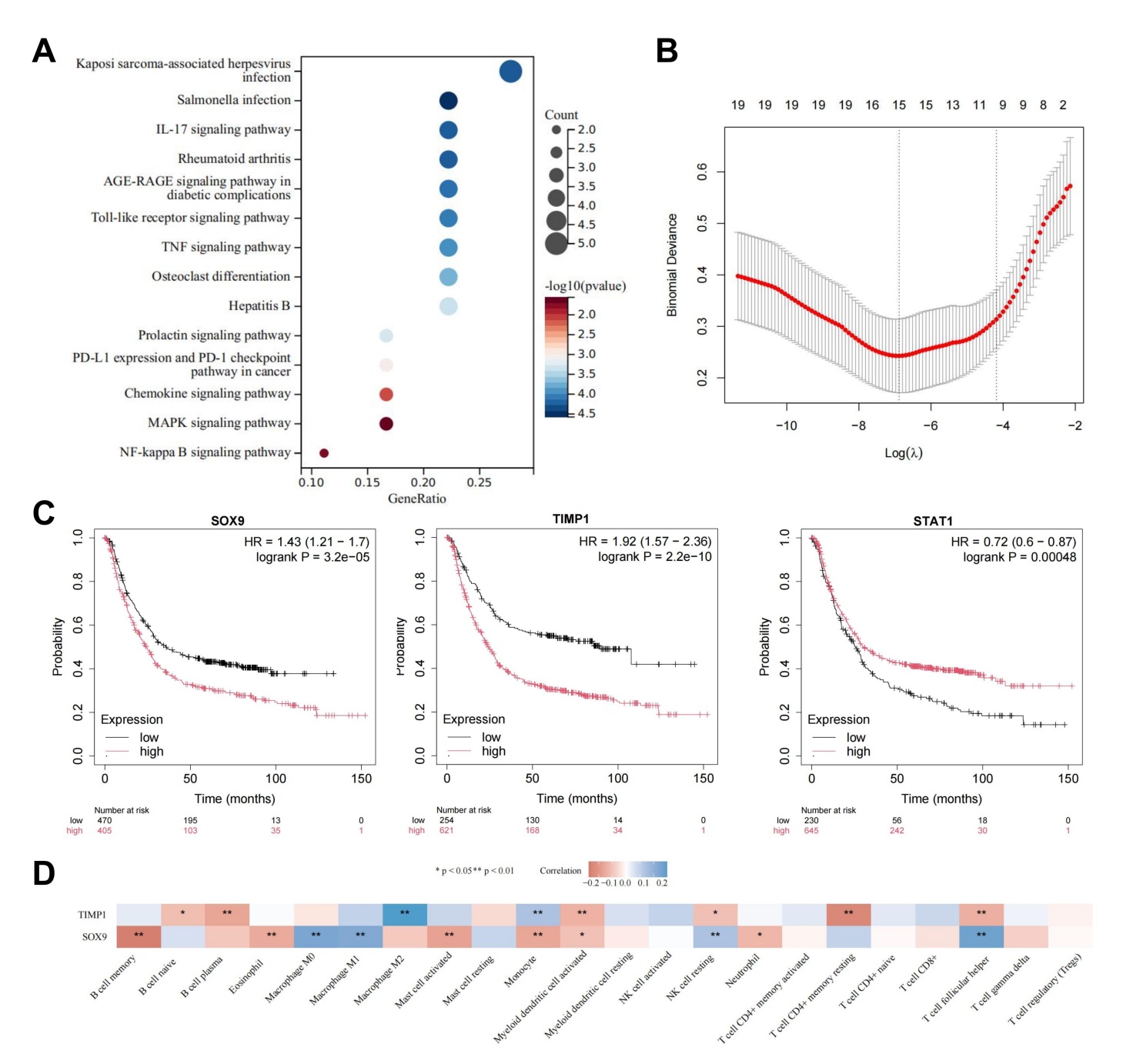
**

**Figure S3. Pathway enrichment analysis of key genes and survival analysis.**

Note: (A) KEGG pathway enrichment analysis results for 21 intersecting differential genes; (B) LASSO regression analysis showing the optimal Lambda value for selecting key genes; (C) Kaplan-Meier survival analysis illustrating the correlation between the expression of *SOX9*, *TIMP1*, and *STAT1* and patient survival; (D) Heatmap of the correlation between *SOX9* and *TIMP1* expression and immune cell infiltration, **p* < 0.05, ***p* < 0.01, GC tumor tissue samples: n=375.

**
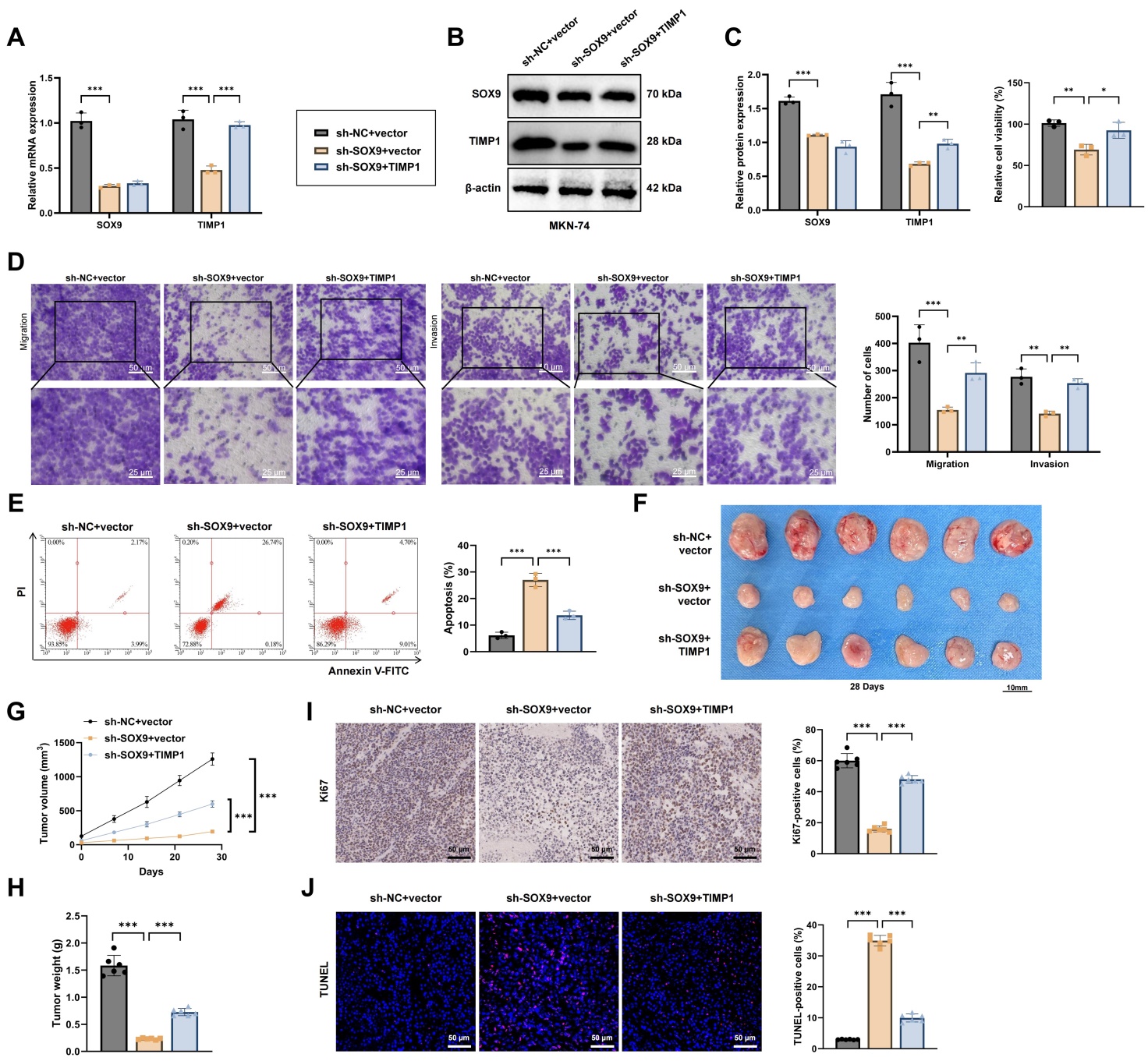
**

**Figure S4. Knockdown of SOX9 through *TIMP1* affects the proliferation, migration, invasion, and apoptosis of MKN-74 cells.**

Note: (A-B) RT-qPCR and Western Blot analysis of SOX9 and *TIMP1* mRNA and protein levels in MKN-74 cells after knockdown of SOX9 or combined overexpression with *TIMP1*; (C) CCK-8 assay to assess cell viability; (D) Transwell assay to evaluate cell migration and invasion (Scale bars=25, 50 μm); (E) Flow cytometry analysis of cell apoptosis; (F) Anatomical diagrams of xenografts in nude mice 4 weeks post-transplant; (G) Line graph showing changes in xenograft volume over time in different groups; (H) Bar graph of xenograft weights 4 weeks post-transplant in different groups; (I-J) Immunohistochemical staining for Ki67 and TUNEL staining assessing cell proliferation and apoptosis in tumor tissues (Scale bars=50 μm). Data presented as mean ± SD, cell experiments independently repeated three times, animal experiments with six nude mice per group, **p*<0.05, ***p*<0.01, ****p*<0.001.

**
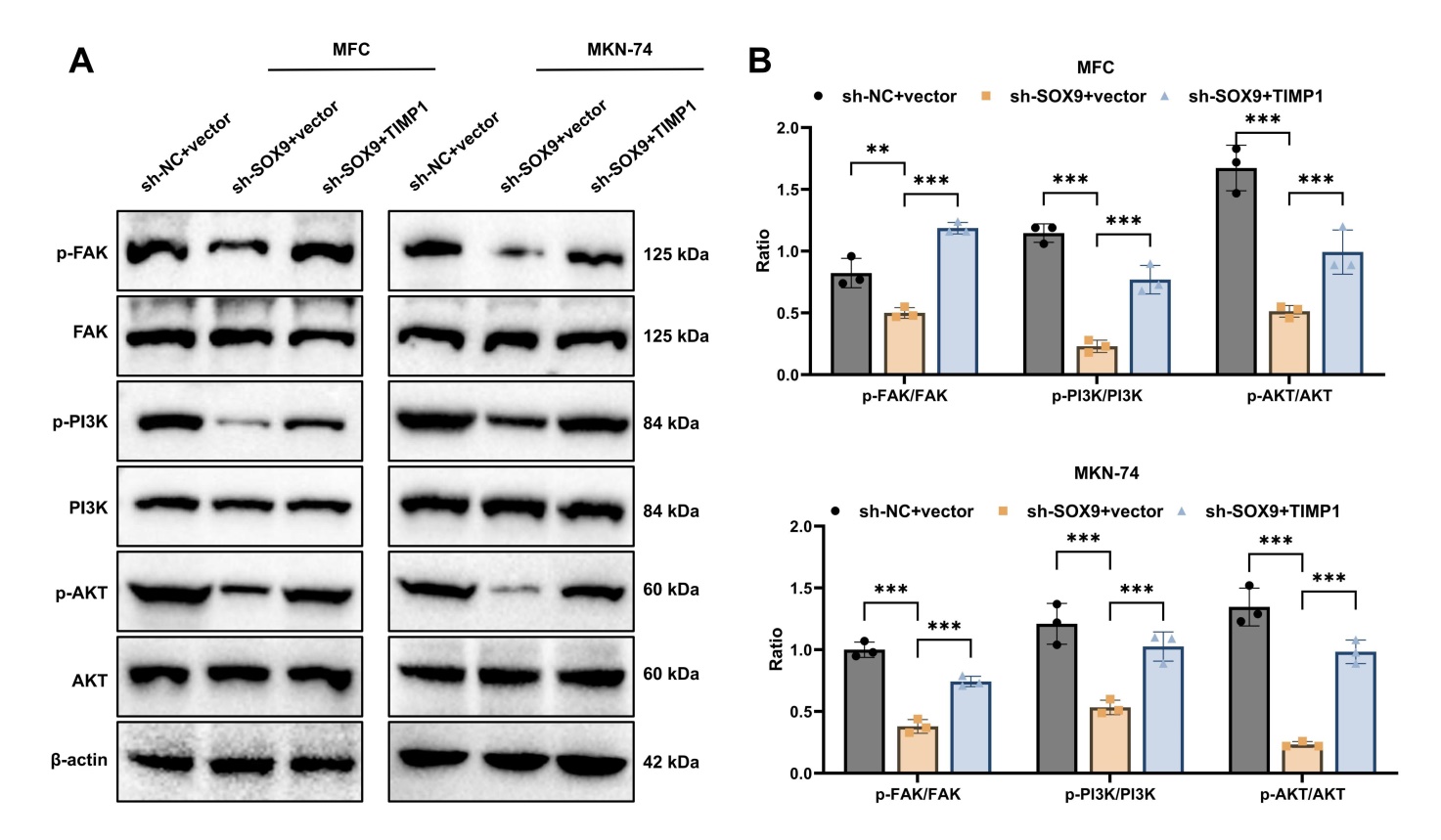
**

**Figure S5. Regulation of FAK/PI3K/AKT signaling pathway proteins by SOX9 knockdown through *TIMP1*.**

**Note: (A) Western Blot analysis of FAK, PI3K, AKT, and their phosphorylated forms (p-FAK, p-PI3K, p-AKT) in MFC and MKN-74 cells post-knockdown of SOX9 or combined overexpression with TIMP1; (B) Quantitative analysis of the ratio of phosphorylated proteins to total proteins. Data presented as mean ± SD, experiments independently repeated three times, **p*<0.05, ***p*<0.01, ****p*<0.001.**

**
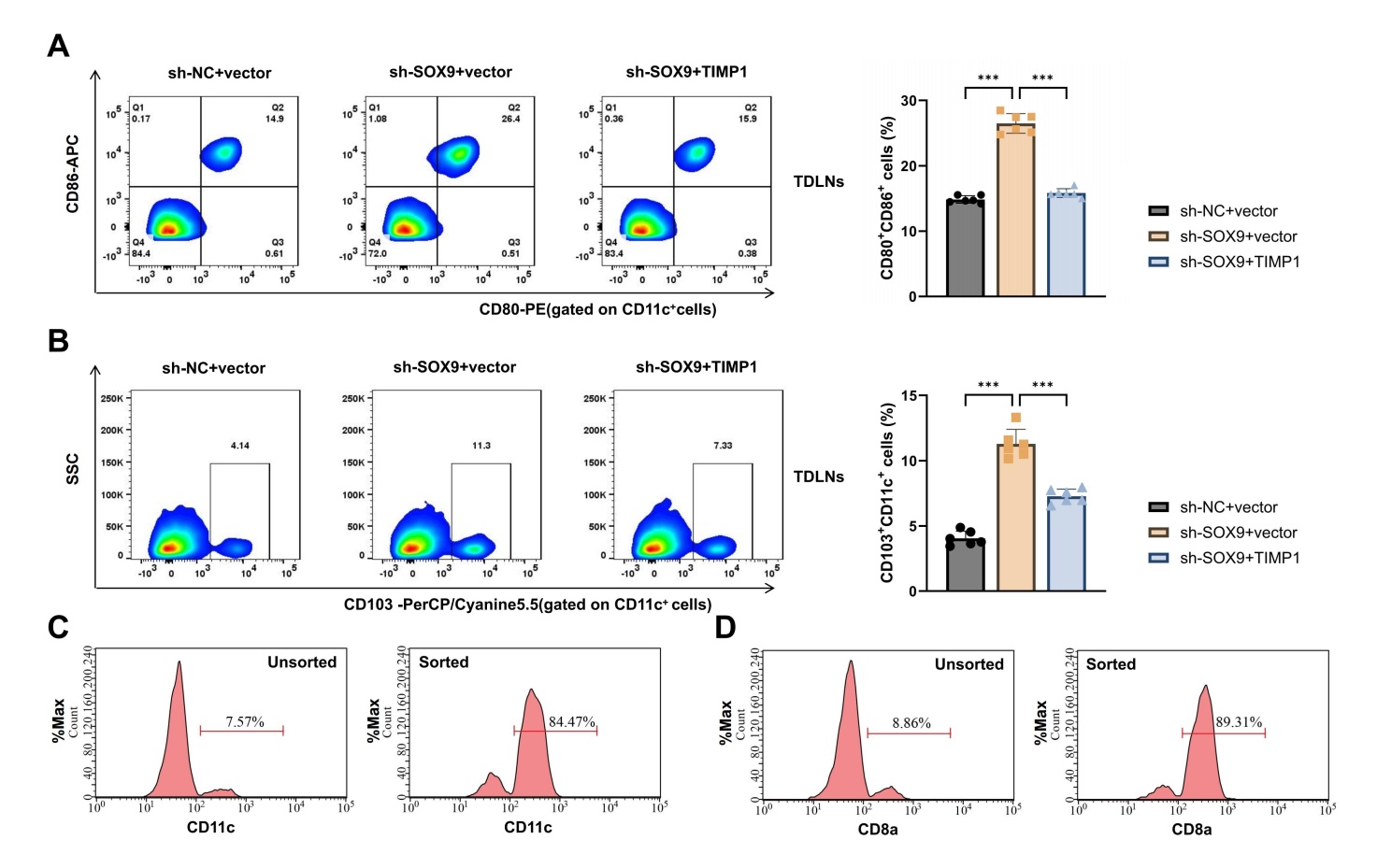
**

**Figure S6. Regulation of DC maturation in TDLNs by SOX9/TIMP1 and cell purity validation in *in vitro* experiments.**

**Note: (A-B) Flow cytometry analysis of the proportions of CD80^+^CD86^+^ and CD103^+^ DCs in subcutaneous xenograft model mouse TDLNs; (C) Flow cytometry analysis of the purity of CD11c^+^ DCs cultured; (D) Flow cytometry analysis of the purity of isolated CD8^+^T cells. Animal experiments with six mice per group, **p*<0.05, ****p*<0.001.**

**
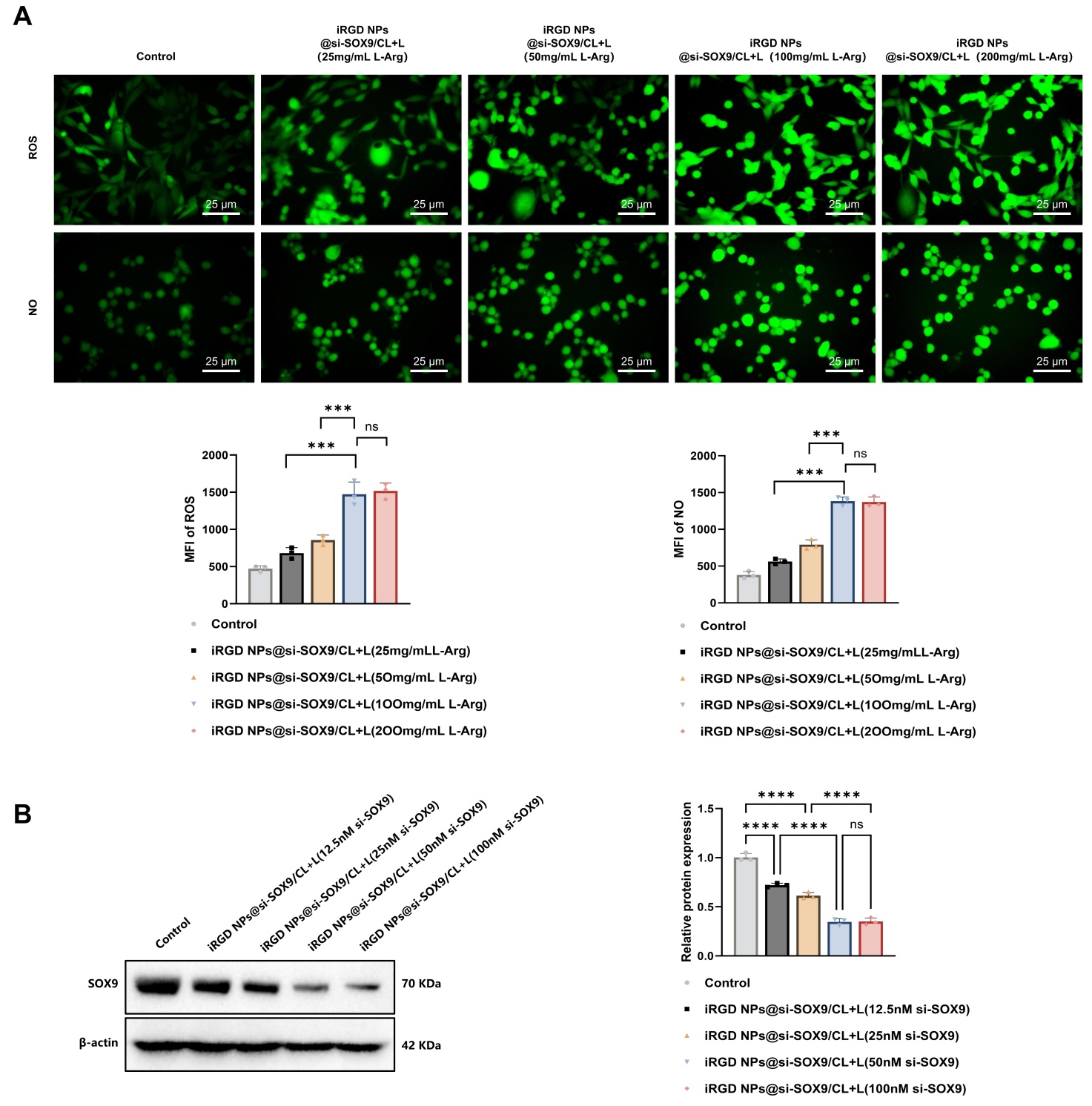
**

**Figure S7. Effects of different nanoparticle components on hypoxia relief or gene silencing efficiency.**

**Note: (A) Intracellular ROS and NO generation were measured under different L-Arg concentrations following iRGD NPs@si-SOX9/CL+L treatment using DCFH-DA and DAF-FM DA probes (Scale bars=25 μm); (B) Western blot analysis of SOX9 protein expression in cells treated with iRGD NPs@si-SOX9/CL+L under different si-SOX9 concentrations. Data are presented as mean ± SD from three independent experiments. ns, not significant; **P*<0.05, ***P*<0.01, ****P*<0.001.**

**
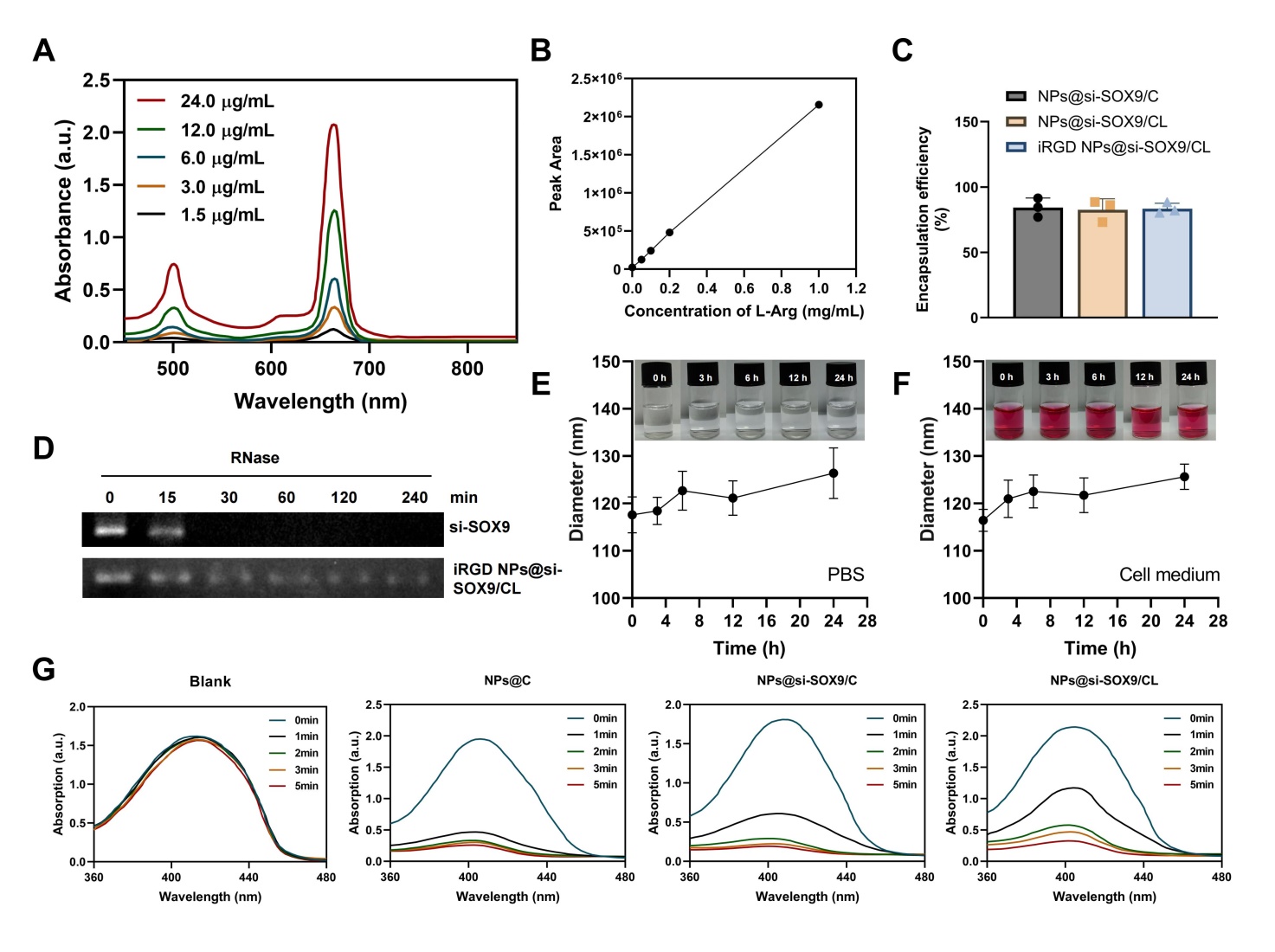
**

**Figure S8. Physicochemical properties and stability of iRGD NPs@si-SOX9/CL.**

**Note: (A) UV-visible spectra and standard curves of free Ce6 in chloroform at different concentrations; (B) HPLC standard calibration curve for L-Arg; (C) siRNA EE% of NPs@si-SOX9/C, NPs@si-SOX9/CL, and iRGD NPs@si-SOX9/CL; (D) RNase protection assay showing significant protection of siRNA from degradation by iRGD NPs@si-SOX9/CL; (E-F) DLS analysis of particle size changes of iRGD NPs@si-SOX9/CL incubated in PBS and DMEM with 10% serum over time; (G) DPBF assay for the generation of ^1^O_2_ by Blank, NPs@C, NPs@si-SOX9/C, and NPs@si-SOX9/CL.**

**
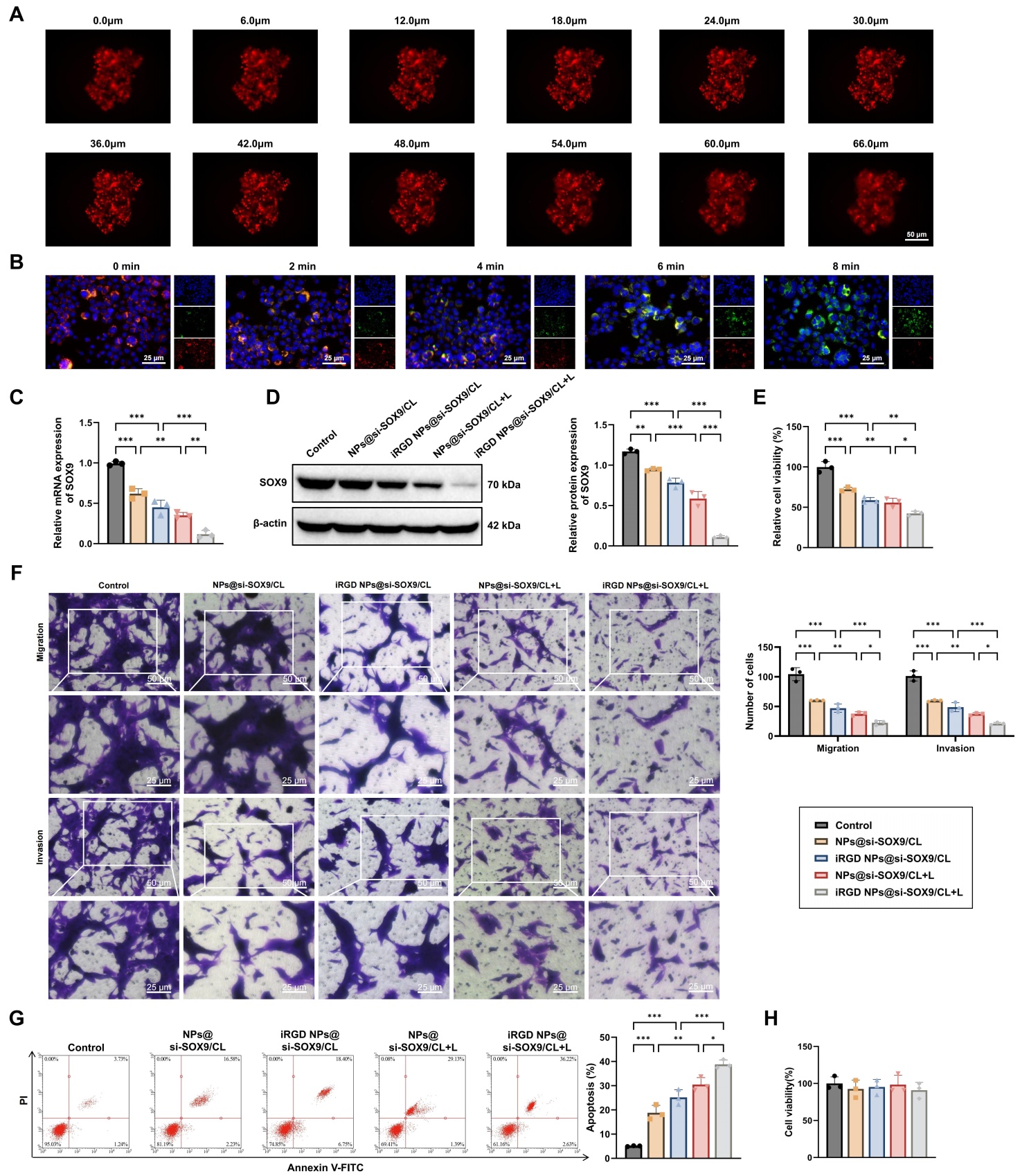
**

**Figure S9. Intracellular behavior and antitumor activity of iRGD NPs@si-SOX9/CL.**

**Note: (A) CLSM imaging showing effective distribution of iRGD NPs@si-SOX9/CL within MCTS; (B) CLSM observation of the release process of iRGD NPs@si-SOX9/CL from endosomes post-NIR irradiation (Scale bars=25 μm); (C-D) RT-qPCR and Western Blot analysis of SOX9 mRNA and protein expression in cells treated with NPs@si-SOX9/CL or iRGD NPs@si-SOX9/CL, with and without NIR irradiation; (E-F) CCK-8 (E) and Transwell (F) assays assessing the viability, migration, and invasion capabilities of various groups of MFC cells (Scale bars=50 μm); (G) Flow cytometry analysis of apoptosis in MFC cells from each group. (H) CCK-8 assay of GES-1 cell viability. Cell experiments independently repeated three times, ns, not significant; **p*<0.05, ***p*<0.01, ****p*<0.001.**

**
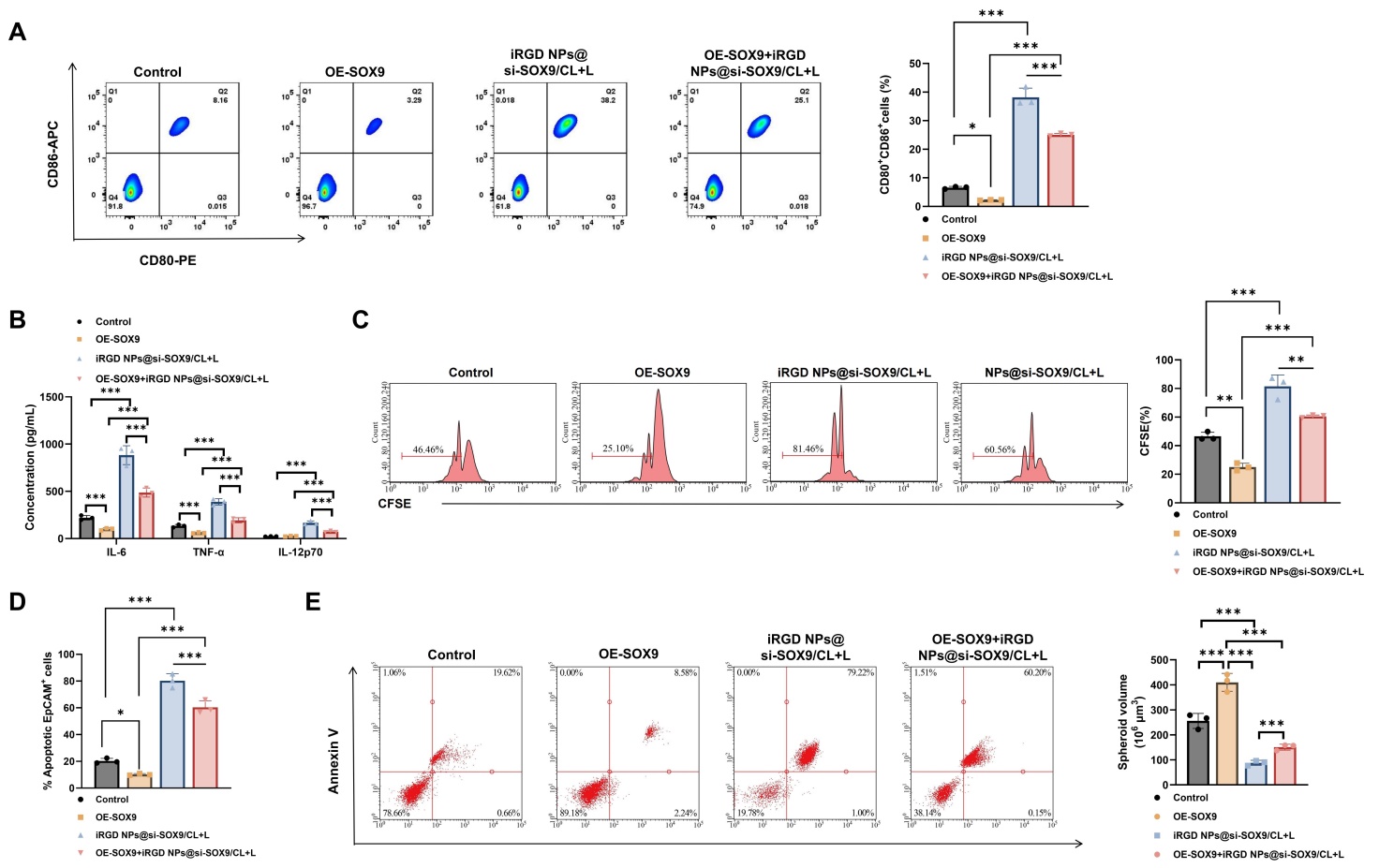
**

**Figure S10. Inhibition of TIMP1 by iRGD NPs@si-SOX9/CL is dependent on SOX9**

**Note: (A) Flow cytometry analysis of the proportion of CD80⁺CD86⁺ DCs after co-culture with treated MFC cells; (B) ELISA measurement of IL-6, TNF-α, and IL-12p70 levels in co-culture supernatants; (C) CFSE-based analysis of CD8⁺ T-cell proliferation after co-culture with DCs; (D) MCTS volume changes across groups; (E) Flow cytometry analysis of tumor cell apoptosis within MCTS. Data are presented as mean ± SD from three independent experiments. **p*<0.05, ***p*<0.01, ****p*<0.001.**

**
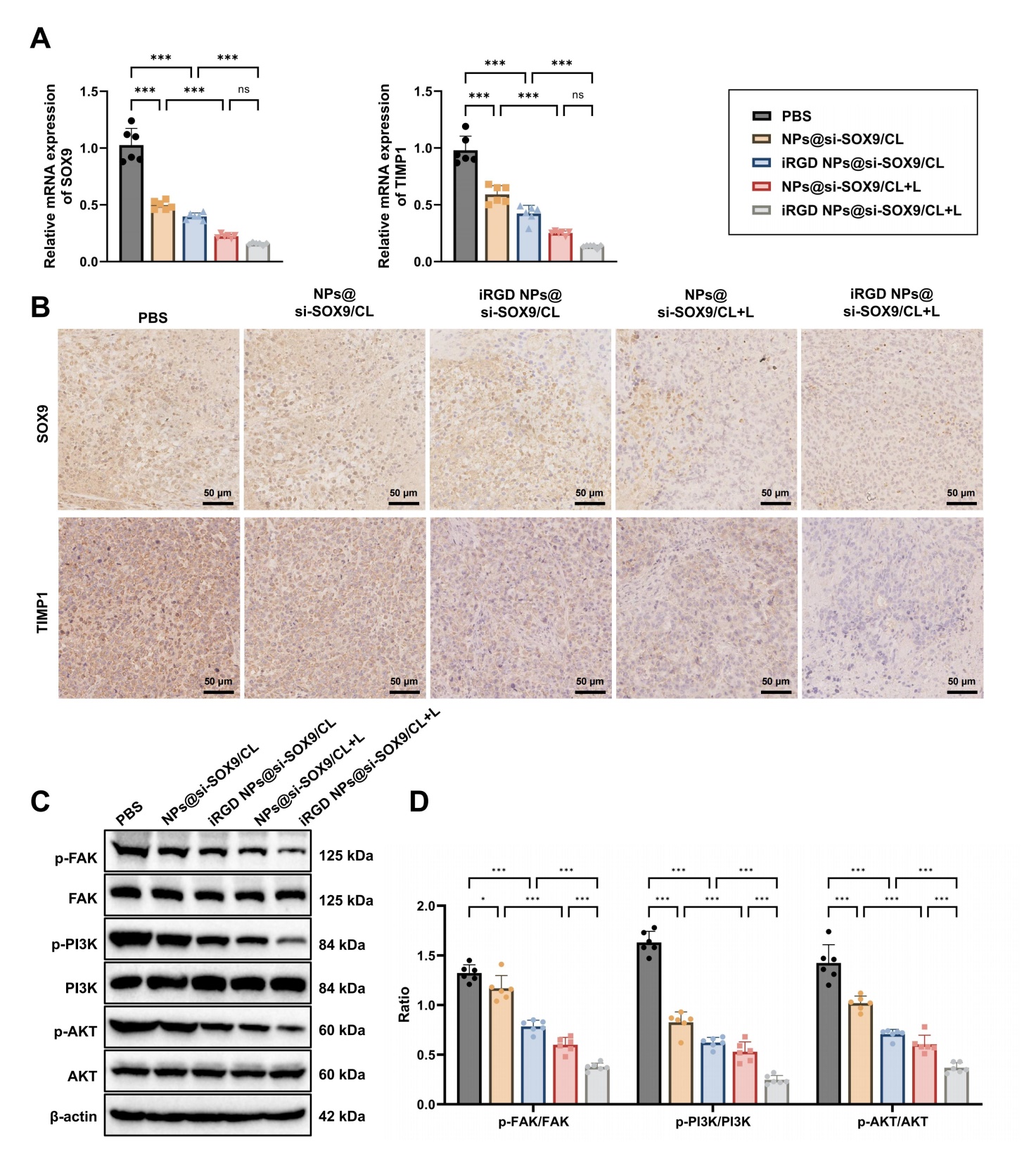
**

**Figure S11. Expression changes of SOX9/TIMP1/PI3K pathway-related factors.**

Note: (A-B) RT-qPCR and immunohistochemical staining to measure the expression of SOX9 and TIMP1 in tumor tissues (Scale bars=50 μm); (C-D) Western Blot analysis to detect and quantify the phosphorylation levels of FAK, PI3K, and AKT in tumor tissues. Data presented as mean ± SD, with six mice per group, **p*<0.05, ***p*<0.01, ****p*<0.001.

**
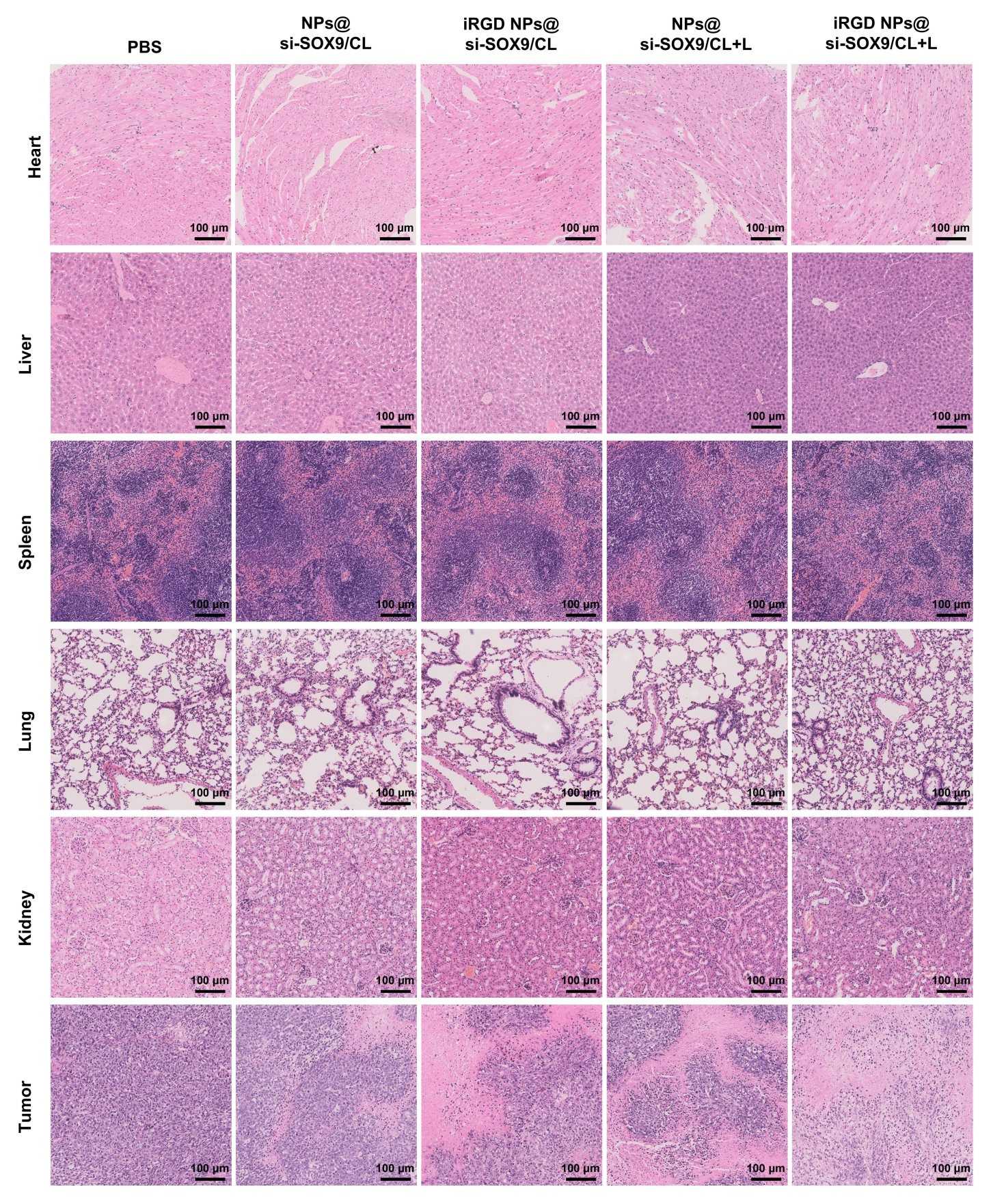
**

**Figure S12. H&E staining of major organs in various mouse groups.**

Note: Scale bars=100 μm.

**
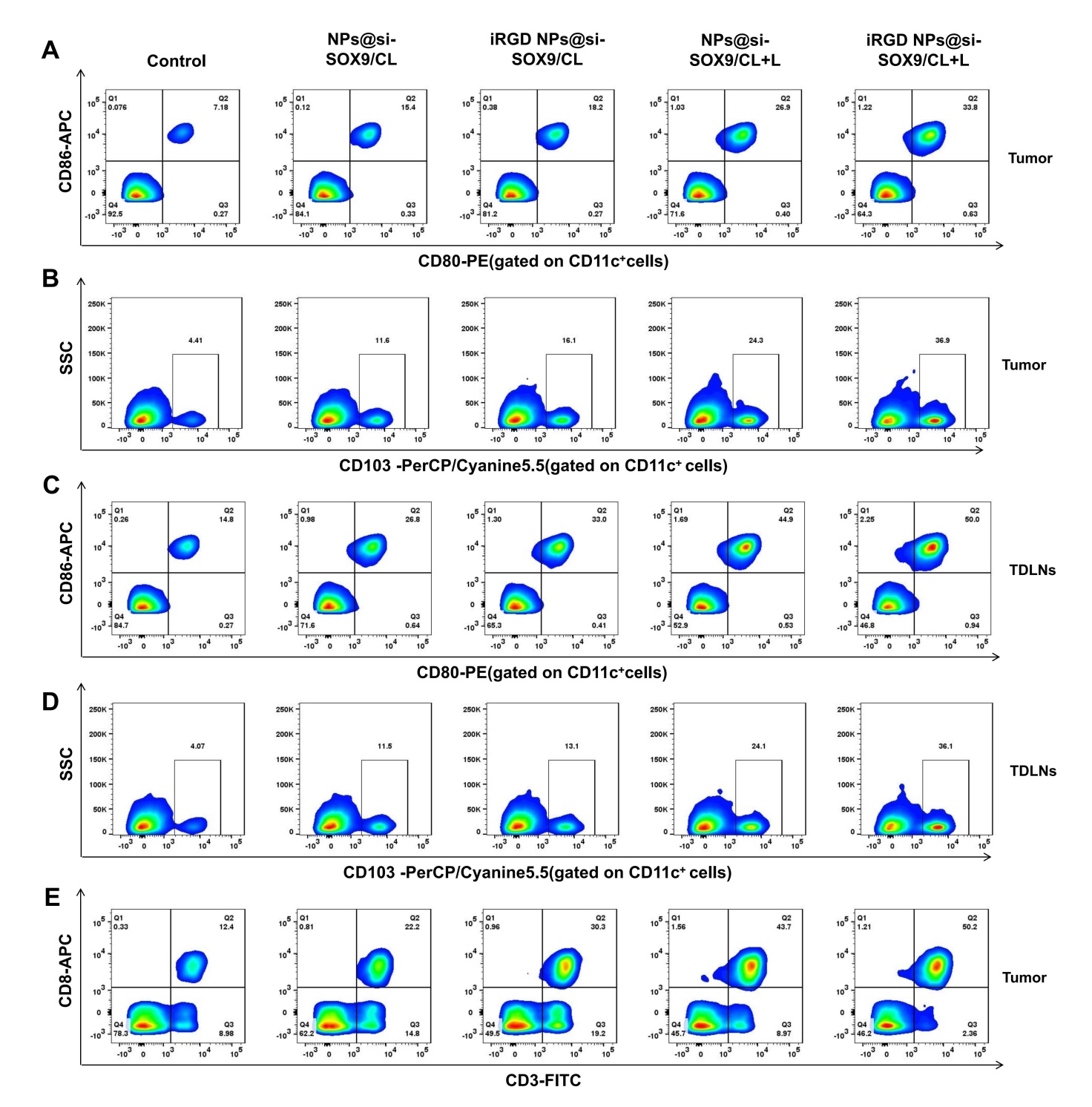
**

**Figure S13. Impact of iRGD NPs@si-SOX9/CL on dendritic and T cell activation in tumor tissues and TDLNs.**

Note: (A-B) Flow cytometry analysis of the proportions of CD80^+^CD86^+^ and CD103^+^ DCs in tumor tissues; (C-D) Flow cytometry analysis of the proportions of CD80^+^CD86^+^ and CD103^+^ DCs in TDLNs; (E) Flow cytometry analysis of CD8^+^T cell infiltration in tumor tissues. Each group consists of six mice.

**
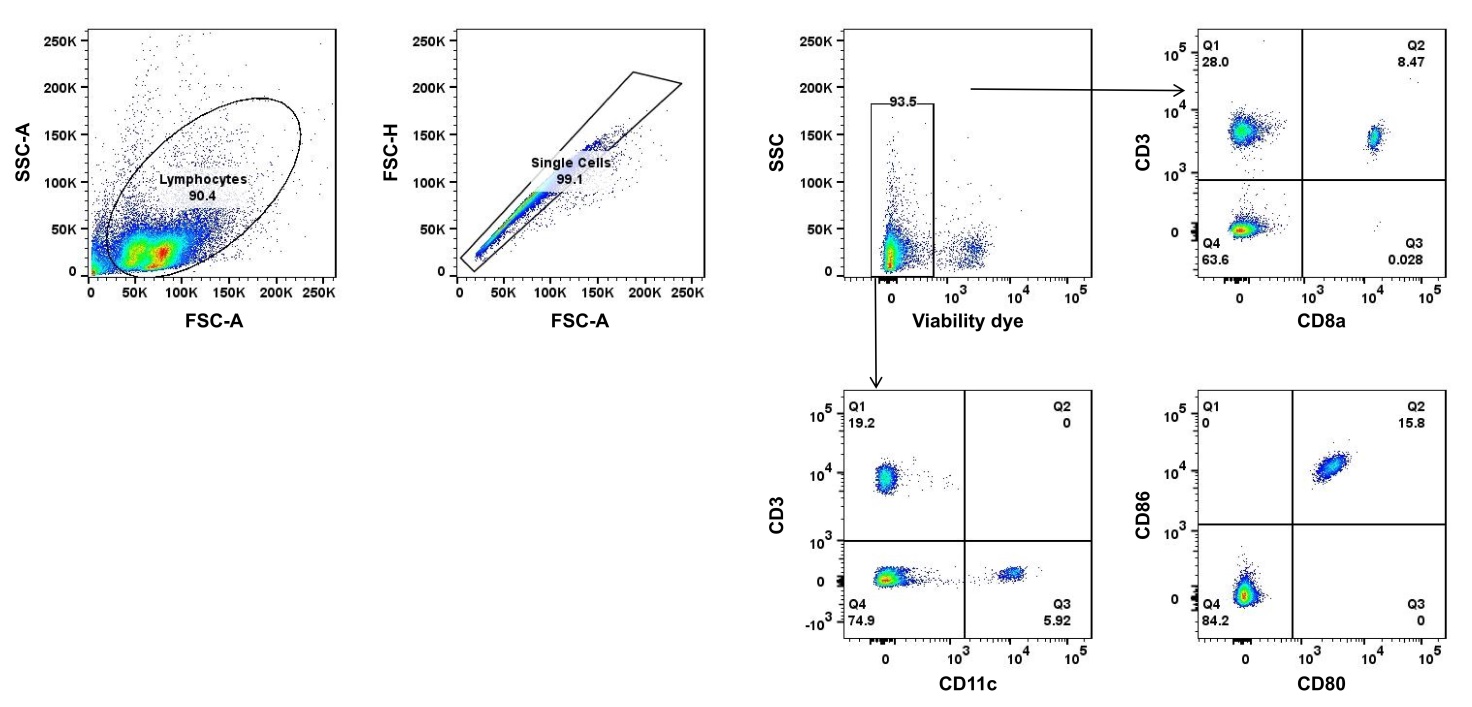
**

**Figure S14. Flow cytometry gating strategy.**
