# Supplementary material for: Targeting the SOX9/TIMP1 Axis with iRGD‐Conjugated Nanoplatform Enhances Dendritic Cell Function and Photodynamic Immunotherapy in Gastric Cancer
Source: Adv Sci (Weinh). 2025 Nov 21;13(3):e10500. doi: 10.1002/advs.202510500 (PMC12806509; doi:10.1002/advs.202510500)
Supplement: Supplementary file 3 — Supporting Information [file ADVS-13-e10500-s003.docx]

**Table S1. Binding site of SOX9 to the TIMP1 promoter region.**

| **Name** | **Score** | **Relative score** | **Sequence ID** | **Star** | **End** | **Strand** | **Predicted sequence** |
| --- | --- | --- | --- | --- | --- | --- | --- |
| MA0077.1.SOX9 | 8.12682 | 0.860299148 | TIMP1 | 1572 | 1580 | - | CCCTTGTTT |
| MA0077.1.SOX9 | 8.009666 | 0.856936037 | TIMP1 | 860 | 868 | + | TTATTGATC |
| MA0077.2.SOX9 | 7.8210506 | 0.862812023 | TIMP1 | 1573 | 1580 | - | CCCTTGTT |
| MA0077.1.SOX9 | 7.425222 | 0.840158411 | TIMP1 | 1296 | 1304 | - | GCATAGTTT |
| MA0077.2.SOX9 | 7.3774295 | 0.849361192 | TIMP1 | 918 | 925 | + | GCATTGTC |
| MA0077.2.SOX9 | 7.362042 | 0.848894633 | TIMP1 | 860 | 867 | + | TTATTGAT |
| MA0077.1.SOX9 | 7.2406273 | 0.83485926 | TIMP1 | 1609 | 1617 | - | TTATTGTGC |
| MA0077.1.SOX9 | 7.1994457 | 0.833677063 | TIMP1 | 604 | 612 | + | ACATTGTAC |
| MA0077.2.SOX9 | 7.119452 | 0.841539176 | TIMP1 | 1297 | 1304 | - | GCATAGTT |
| MA0077.1.SOX9 | 6.988927 | 0.827633707 | TIMP1 | 918 | 926 | + | GCATTGTCA |
| MA0077.1.SOX9 | 6.9444027 | 0.826355553 | TIMP1 | 504 | 512 | - | CTATTTTTT |
| MA0077.2.SOX9 | 6.6386333 | 0.826960494 | TIMP1 | 505 | 512 | - | CTATTTTT |
| MA0077.2.SOX9 | 6.593003 | 0.825576954 | TIMP1 | 1610 | 1617 | - | TTATTGTG |
| MA0077.2.SOX9 | 6.5518208 | 0.824328292 | TIMP1 | 604 | 611 | + | ACATTGTA |
| MA0077.2.SOX9 | 6.215207 | 0.814121986 | TIMP1 | 851 | 858 | + | CAATTATT |
| MA0077.2.SOX9 | 5.807081 | 0.801747391 | TIMP1 | 700 | 707 | + | TTTTTGTT |

**Table S2. RT-qPCR primer sequences.**

| **Gene** | **primer sequences (5’-3’)** |
| --- | --- |
| SOX9 (human) | F AGCGAACGCACATCAAGAC |
|  | R CTGTAGGCGATCTGTTGGGG |
| TIMP1 (human) | F CTTCTGCAATTCCGACCTCGT |
|  | R ACGCTGGTATAAGGTGGTCTG |
| SOX9 (mouse) | F GAGCCGGATCTGAAGAGGGA |
|  | R GCTTGACGTGTGGCTTGTTC |
| TIMP1 (mouse) | F CGAGACCACCTTATACCAGCG |
|  | R ATGACTGGGGTGTAGGCGTA |
| β-actin (human) | F AATTTGCGTGTGGCTCCCGAGG |
|  | R GGATAGCACAGCCTGGATAGCA |
| β-actin (mouse) | F GTGACGTTGACATCCGTAAAGA |
|  | R GCCGGACTCATCGTACTCC |

Note: F: Forward, R: Reverse.
